# Transcriptomic analysis of mitochondrial TFAM depletion changing cell morphology and proliferation

**DOI:** 10.1038/s41598-017-18064-9

**Published:** 2017-12-19

**Authors:** Woo Rin Lee, Heeju Na, Seon Woo Lee, Won-Jun Lim, Namshin Kim, J. Eugene Lee, Changwon Kang

**Affiliations:** 1 Department of Biological Sciences, Korea Advanced Institute of Science and Technology, Daejeon, 34141 Korea; 2 Center for Bioanalysis, Korea Research Institute of Standards and Science, Daejeon, 34113 Korea; 3 Genome Editing Research Center, Korea Research Institute of Bioscience and Biotechnology, Daejeon, 34141 Korea; 4 Department of Bioinformatics, KRIBB School of Bioscience, Korea University of Science and Technology, Daejeon, 34141 Korea

## Abstract

Human mitochondrial transcription factor A (TFAM) has been implicated in promoting tumor growth and invasion. TFAM activates mitochondrial DNA (mtDNA) transcription, and affects nuclear gene expression through mitochondrial retrograde signaling. In this study, we investigated the effects of TFAM depletion on the morphology and transcriptome of MKN45 gastric cancer cells. Morphology alteration became visible at 12 h after TFAM knockdown: the proportion of growth-arrested polygonal cells versus oval-shaped cells increased, reaching a half-maximum at 24 h and a near-maximum at 36 h. TFAM knockdown upregulated four genes and downregulated six genes by more than threefold at 24 h and similarly at 48 h. Among them, the knockdown of CFAP65 (cilia and flagella associated protein 65) or PCK1 (cytoplasmic phosphoenolpyruvate carboxykinase) rescued the effects of TFAM depletion on cell morphology and proliferation. PCK1 was found to act downstream of CFAP65 in calcium-mediated retrograde signaling. Furthermore, mtDNA depletion by 2′,3′-dideoxycytidine was sufficient for induction of *CFAP65* and *PCK1* expression and inhibition of cell proliferation, but oxidative phosphorylation blockade or mitochondrial membrane potential depolarization was not. Thus, the TFAM–mtDNA–calcium–CFAP65–PCK1 axis participates in mitochondrial retrograde signaling, affecting tumor cell differentiation and proliferation.

## Introduction

Human mitochondrial transcription factor A, encoded by the nuclear gene *TFAM*, plays essential roles in the transcription, replication, and packaging of circular mitochondrial genome DNA (mtDNA) into nucleoids and has critical roles in mitochondrial biogenesis. TFAM regulates mtDNA copy number by maintaining the stability of mtDNA^1^, and mitochondrial biogenesis is regulated through the interaction of TFAM with mtDNA^2^.

Genome-wide transcriptomic profiles of cancerous and normal tissues can provide useful information about the molecular mechanisms of cancer initiation and progression. According to the ONCOMINE database of cancer microarray assays, TFAM is upregulated in many types of cancer tissues^3^. TFAM is a key molecule in carcinogenesis, owing to its involvement in cell proliferation and invasion as well as its interference with apoptosis^4,5^.

In this study, we analyzed the effects of TFAM depletion on gastric cancer MKN45 cells to gain insight into the functional role of TFAM in human cancer. Interestingly, TFAM depletion was found to decrease the rate of cell division as cells organize into polarized growth-arrested polygonal shaped colonies, which is a common phenotype observed in non-malignant cancer cells^6^.

To understand the mechanism underlying this morphological shift, we first analyzed the transcriptome of TFAM-depleted MKN45 cells using next-generation RNA sequencing (RNA-Seq). Next, we assessed the functional roles of the ten most differentially expressed genes (DEGs) in promoting the phenotypic changes. In addition, we investigated how the expression of the DEGs was altered in response to mitochondrial retrograde signaling.

Furthermore, to understand the clinical significance of *TFAM* expression in cancer, we tested whether *TFAM* polymorphisms were associated with susceptibility to gastric cancer. Finally, we characterized a novel TFAM downstream pathway that may provide mechanistic insight into cell differentiation and proliferation, and contribute to the rational development of new prognostic and therapeutic tools for cancer treatment.

## Results

### Effects of TFAM knockdown on cell proliferation

To understand the functional role of TFAM in cancer, we depleted TFAM in the MKN45 cell line, which has the highest level of TFAM mRNA among the eleven gastric cancer cell lines in the GENT database^7^. MKN45 cells were transfected with two different small interfering RNAs (siRNAs) against TFAM, siTFAM#1 (HSS144251 from Invitrogen, Carlsbad, CA), and siTFAM#2 (HSS144250).

**Figure 1:**
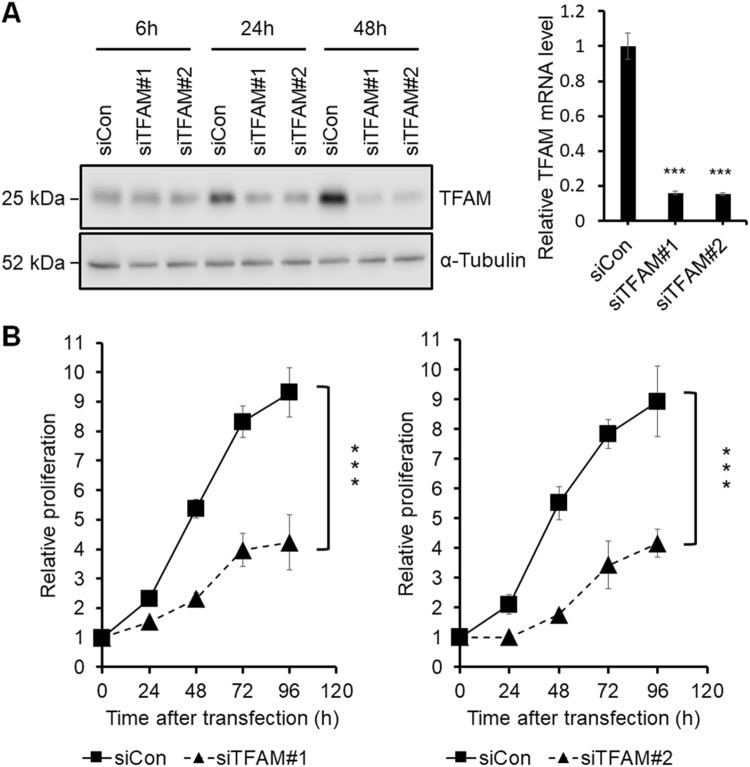
Screening of DEGs related to the TFAM knockdown effects on the proliferation of MKN45 cells. **(A)** Left panel: western blot analysis showing the protein levels of siTFAM#1-, siTFAM#2-, and siCon-transfected MKN45 cells at 6 h, 24 h, and 48 h after transfection. Right panel: qPCR analysis of TFAM mRNA levels at 24 h after transfection (*n* = 3, mean ± SD). Student’s t test, ***P < 0.001. Full-length blots are presented in Supplementary Figure S1. **(B)** MTT assay of siTFAM#1-, siTFAM#2-, and siCon-transfected MKN45. *A*_570 nm_ was measured at 0, 24, 48, 72, and 96 h after transfection (*n* = 3, mean ± SD). Student’s t test, ***P < 0.001.

The resulting knockdown of TFAM at the protein and mRNA levels was confirmed by using western blotting and quantitative real-time polymerase chain reaction (qPCR), respectively (Fig. 1A). TFAM depletion has previously been shown to decrease cell proliferation in esophageal, arsenical skin, and prostate cancers^8–10^. Likewise, in this study, TFAM knockdown using either siTFAM#1 or siTFAM#2 decreased the proliferation of MKN45 gastric cancer cells (Fig. 1B).

To assess the extent to which cell death caused the decrease in proliferation, we counted necrotic and apoptotic cells using fluorescence-activated cell sorting analysis. TFAM depletion had no effect on cell death (data not shown), thus suggesting that the decreased proliferation was probably due to cell cycle retardation or arrest, in agreement with a previous finding that TFAM depletion induces G1 arrest in a non-small cell lung cancer cell line^5^.

### Effects of TFAM knockdown on cell morphology

The MKN45 cell line, derived from a poorly differentiated adenocarcinoma of the stomach, has characteristics of both ordinary gastric mucosa and intestinal metaplastic mucosa^11^. MKN45 cells grow partly as adherent polygonal epithelial cells and partly as piled-up, oval-shaped cells in two-dimensional cultures.

**Figure 2:**
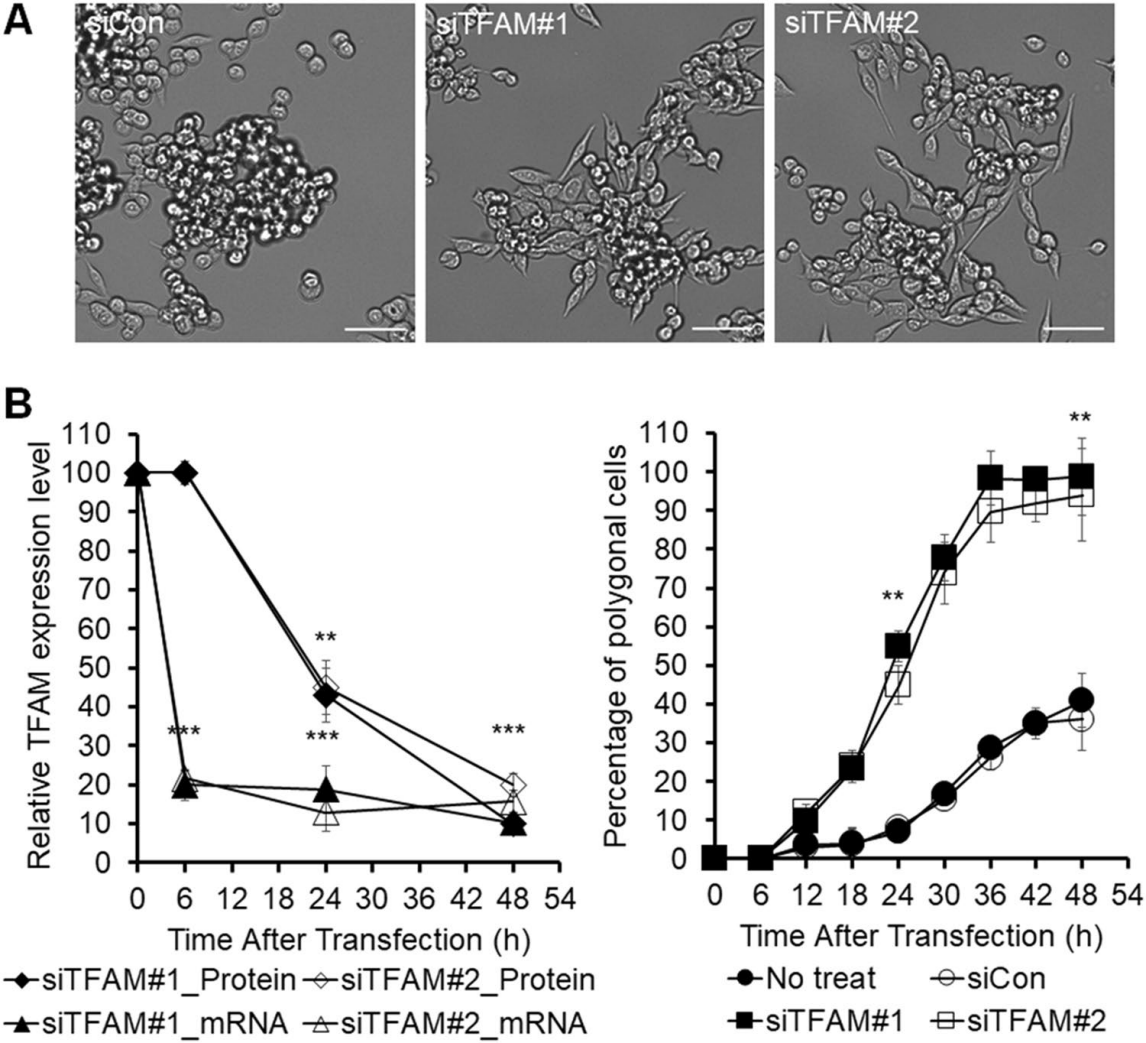
Screening of DEGs related to the TFAM knockdown effects on the morphology of MKN45 cells. **(A)** Representative bright-field images of morphological changes in TFAM-knockdown MKN45. The scale bar represents 50 µm. **(B)** RNA-Seq samples were prepared at 0, 6, 24, and 48 h after transfection. Left panel: time-course analysis of the TFAM mRNA and protein levels. In qPCR, relative mRNA levels were normalized to GAPDH. Relative protein levels were normalized to α-tubulin. Right panel: quantification of polygonal cells of siRNA-transfected and control MKN45 (*n* = 3, mean ± SD). Among the total cells counted with Image-Pro Plus, polygonal cells were distinguished from oval cells using the values of area and roundness. Student’s t-test, **P < 0.01, ***P < 0.001.

TFAM knockdown increased the percentage of adherent polygonal cells (Fig. 2A). Videos of this change in the morphology of MKN45 cells were recorded for 96 h after siRNA transfection by using time-lapse live-cell imaging (Supplementary Video S1), and the polygonal and oval-shaped cells were counted every 6 h up to 48 h (Fig. 2B).

At 6 h after siRNA transfection, TFAM mRNA had already decreased to its minimum level, but the TFAM protein abundance and cell morphology were not yet changed. At 12 h, a morphological shift from oval-shaped to polygonal cells became visible. The percentage of polygonal cells reached 55% at 24 h, 98% at 36 h, and 99% at 48 h (Fig. 2B).

TFAM protein abundance decreased substantially to 43% at 24 h and almost maximally to 10% at 48 h. Thus, TFAM protein abundance was inversely correlated with the polygonal cell percentage. Accordingly, TFAM depletion in MKN45 cells changed the cell morphology, increasing the percentage of polygonal cells.

### Transcriptome analysis using RNA-Seq and qPCR

To gain insight into the mechanism underlying the TFAM depletion-induced changes in cell morphology and proliferation, we sought to identify the genes whose expression is affected by TFAM depletion. To this end, transcriptomes from MKN45 cells were analyzed by time-course RNA-Seq analyses after TFAM depletion.

Total mRNA was extracted from MKN45 cells that were transfected with siTFAM#1, siTFAM#2, or a scrambled siRNA (a negative control) and were sampled at 6, 24, or 48 h after transfection. The three time points were chosen because the cell morphology change and TFAM protein depletion were absent at 6 h, approximately half maximum at 24 h and near maximum at 48 h.

A baseline control cell preparation not subjected to siRNA transfection was additionally analyzed. These ten different cell preparations were each triplicated, thereby generating a total of 30 transcriptome samples that were analyzed using RNA-Seq with a sequencing depth of 55 million paired-end reads per sample, which was several fold deeper than usual transcriptomic analyses.

**Figure 3:**
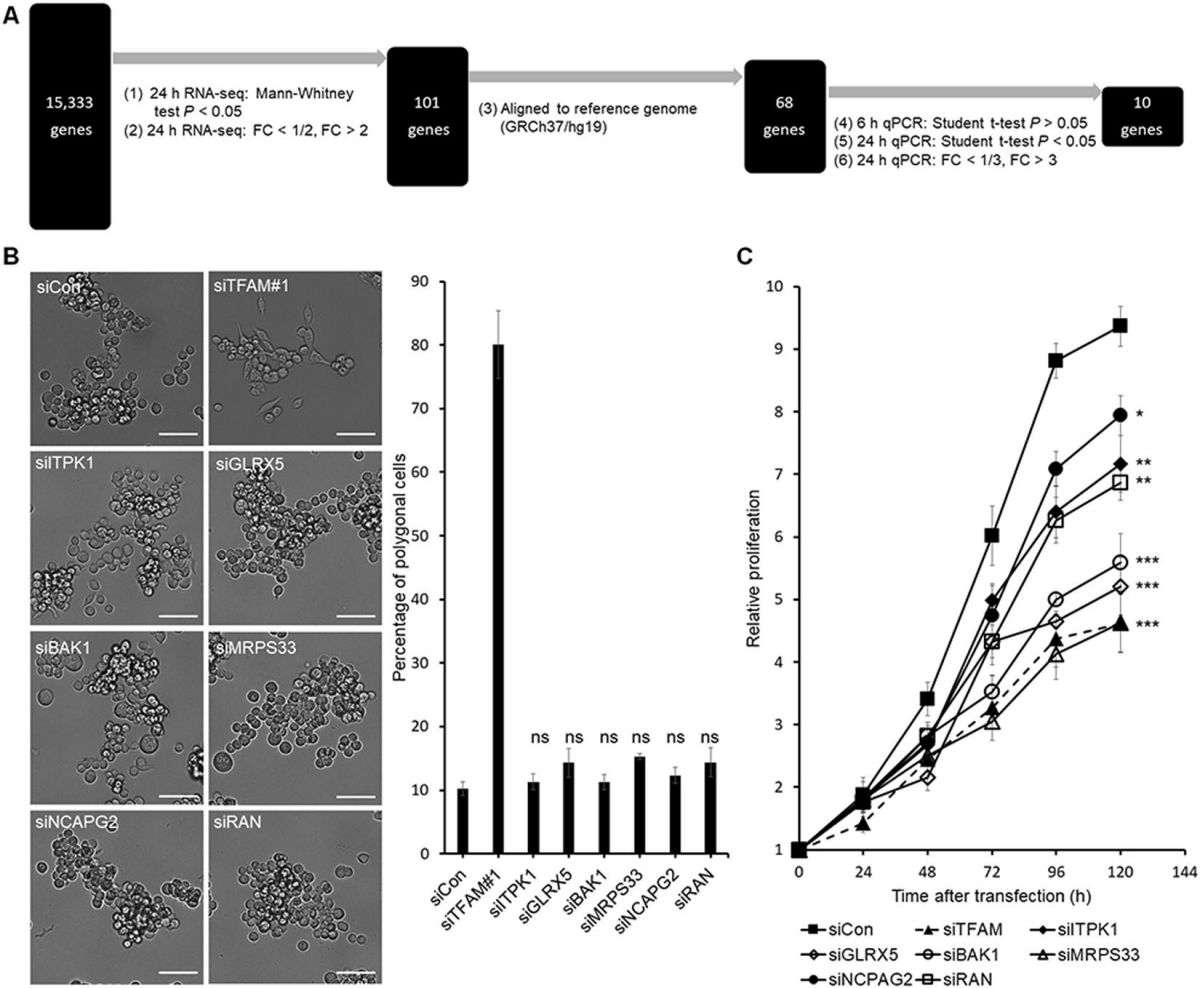
Effects of six downregulated DEGs on the morphology and proliferation of MKN45 cells. **(A)** Scheme of selecting DEGs of TFAM depletion using statistical filtration. From a total of 15,333 genes analyzed in Cuffdiff2, 68 genes were finally selected for quantification with qPCR for the identification of top 10 DEGs. **(B)** Effects of UBLCP1, KRT80, AP1AR, ANKRD13A, LRG1, or TMEM30B depletion on the morphology of MKN45. Left panel: representative bright-field images of MKN45 at 48 h after transfection. The scale bar represents 50 µm. Right panel: percentage of polygonal cells measured using Image-Pro Plus software. **(C)** MTT assay of UBLCP1-, KRT80-, AP1AR-, ANKRD13A-, LRG1-, or TMEM30B-depleted MKN45 cells. *A*_570 nm_ was measured after transfection (*n* = 3, mean ± SD). Student’s t-test, *P < 0.05, **P < 0.01, ***P < 0.001.

Differentially expressed genes (DEGs) of TFAM depletion in MKN45 cells were identified according to the scheme shown in Fig. 3A. Cellular mRNA levels were estimated for a total of 15,333 genes using the Fastqc-TopHat-Cufflinks-CuffDiff2 pipeline. We then calculated the fold change (FC) in the mRNA levels in an experimental sample compared with the siCon sample harvested at the same time point and took an average of the fold changes observed with siTFAM#1 and siTFAM#2 at each time point.

A total of 101 genes showed FC greater than 2 or less than 1/2 and a Mann-Whitney *P* value ≤ 0.05 at 24 h (Supplementary Table S1). Among them, only 68 genes had official names other than XLOC_# in the reference genome GRCh37/hg19. The mRNA levels of these 68 named genes in the 6-, 24-, and 48-h samples treated with siTFAM#1, siTFAM#2, and siCon were individually quantified in triplicate using a total of 1,836 (=68 genes × 3 time points × 3 siRNAs × 3 triplicates) qPCR reactions (Supplementary Table S1).

*TFAM* and six other genes (*UBLCP1*, *KRT80*, *AP1AR*, *ANKRD13A*, *LRG1*, and *TMEM30B*) had significantly changed mRNA levels even at 6 h, when the TFAM protein level had not yet changed. Accordingly, these mRNA changes were unlikely to be related to TFAM-dependent transcription regulation.

Among the remaining 61 genes, ten mRNA levels changed more than threefold (FC > 3 or < 1/3, Student’s t-test *P* < 0.05) at 24 h, and the direction of fold changes were unchanged at 48 h (Table 1). Among these ten DEGs, four were upregulated (*NUPR1*, *EFCAB12*, *PCK1*, and *CFAP65* in descending order of absolute FC values at 24 h), and six were downregulated (*ITPK1*, *GLRX5*, *BAK1*, *MRPS33*, *NCAPG2*, and *RAN* in the same descending order) in response to TFAM depletion.

**Table 1:** Top ten DEGs of TFAM knockdown.

Gene	FC at 6 h	FC at 24 h	FC at 48 h
*NUPR1*	1.1	6.9*	16*
*ITPK1*	1.1	0.17*	0.23*
*EFCAB12*	1.2	5.3*	6.2*
*PCK1*	1.1	4.5*	12*
*CFAP65*	1.5	4.5*	6.1*
*GLRX5*	1.1	0.23*	0.21*
*BAK1*	0.87	0.32*	0.31*
*MRPS33*	0.93	0.32*	0.31*
*NCAPG2*	0.92	0.33*	0.32*
*RAN*	0.88	0.33*	0.42*

The relative fold changes (FC) of mRNA levels after TFAM knockdown were calculated for 68 genes with their GAPDH-normalized ∆Cq values of qPCR. An average of ∆Cq values of the siTFAM#1 and siTFAM#2 cases in triplicate was divided by an average ∆Cq value of the siCon case in triplicate that were measured at 6, 24, and 48 h after siRNA transfection. The genes are listed according to decreasing order of the absolute values of fold changes at 24 h (Student’s t-test, *P < 0.05), and only the top ten genes other than *TFAM* are shown here.

### Effects of DEGs on cell morphology and proliferation

These top ten DEGs were chosen for further functional studies. The six genes downregulated by TFAM depletion were then individually knocked down by using siRNAs against each without TFAM depletion. However, the percentage of polygonal cells did not change in any case (Fig. 3B), although cell proliferation was decreased in every case (Fig. 3C). Accordingly, these six genes were unlikely to be associated with the morphology change.

**Figure 4:**
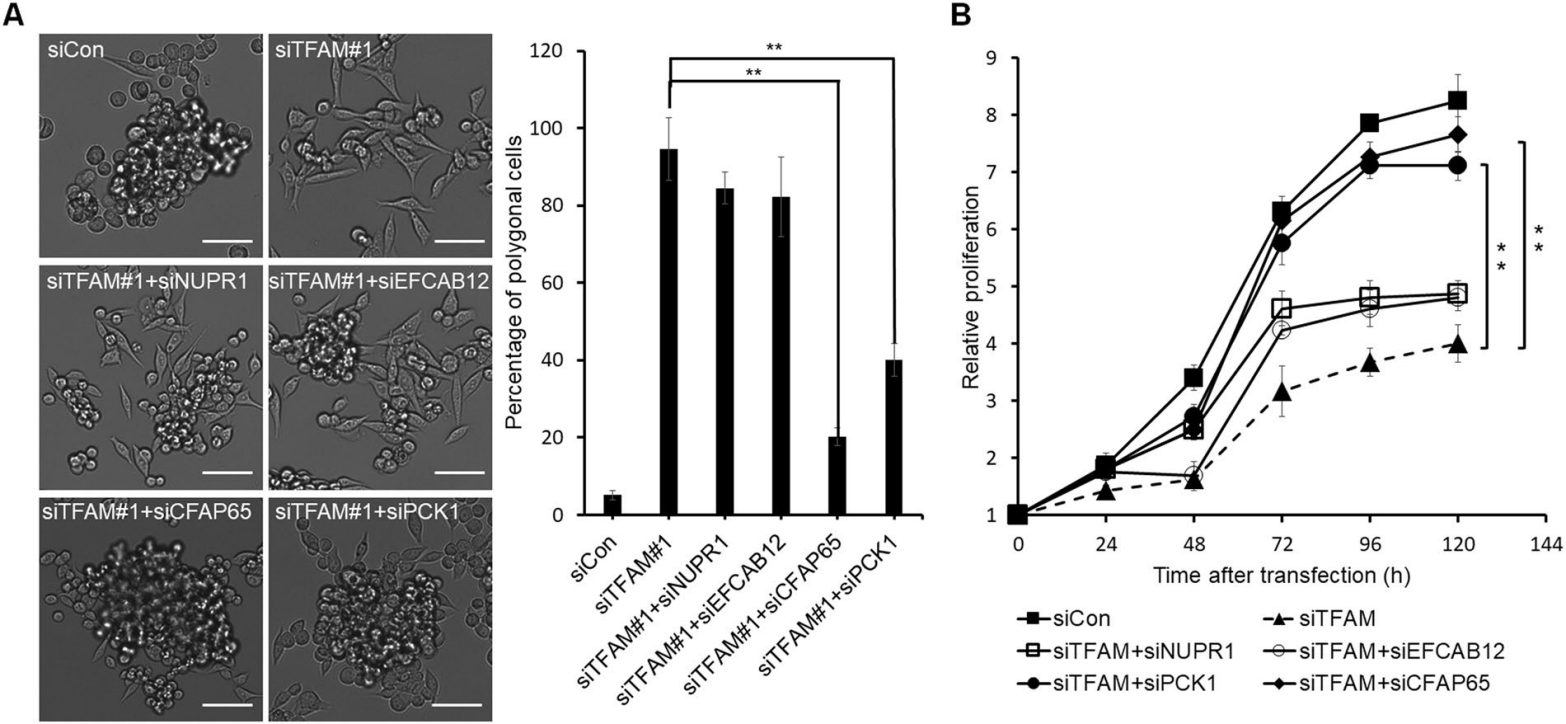
Effects of four upregulated DEGS on the morphology and proliferation of MKN45 cells. **(A)** Effects of CFAP65 or PCK1 depletion on the morphology of TFAM-knockdown MKN45 cells. Left panel: representative bright-field images of MKN45 at 48 h after transfection with siRNA. The scale bar represents 50 µm. Right panel: percentage of polygonal-shape cells measured using Image-Pro Plus software (*n* = 3, mean ± SD). Student’s t test, **P < 0.01. **(B)** Effects of CFAP65 or PCK1 depletion on MTT assay of TFAM-knockdown MKN45 cells. *A*_570 nm_ was measured every 24 h after transfection (*n* = 3, mean ± SD). Student’s t test, **P < 0.01.

Next, the other four genes upregulated by TFAM depletion were individually knocked down by using siRNAs against each of the genes in addition to an anti-TFAM siRNA (siTFAM#1) to compare against the control with anti-TFAM siRNA only. Knockdown of NUPR1 or EFCAB12 did not alter the effect of TFAM knockdown on cell morphology (Fig. 4A) or proliferation (Fig. 4B).

By contrast, knockdown of CFAP65 or PCK1 partially rescued the effects of TFAM knockdown, i.e., increasing the percentage of polygonal cells (Fig. 4A) and decreasing cell proliferation (Fig. 4B). These rescue effects suggested that CFAP65 and PCK1 are involved in the TFAM knockdown-induced changes in morphology and proliferation of MKN45 cells.

### Effects of TFAM knockdown on *CFAP65* and *PCK1* expression

TFAM is essential for maintaining mtDNA copy number and integrity^12^, and TFAM knockdown causes mtDNA depletion^13^ and mitochondrial membrane potential (MMP) depolarization^5^. When TFAM was knocked down in MKN45 cells in this study, the mtDNA copy number measured using qPCR (Supplementary Figure S2A) and mitochondrial potential measured using MitoTracker staining (Supplementary Figure S2B) both decreased, thus confirming that TFAM knockdown also causes mitochondrial dysfunction in MKN45 cells.

Both mtDNA depletion and membrane potential depolarization inhibit mitochondrial uptake of Ca^2+^, thereby elevating the cytoplasmic Ca^2+^ level and activating calcineurin-mediated mitochondrial retrograde signaling to the nucleus^14,15^. mtDNA depletion and membrane potential depolarization additionally stimulate the production of reactive oxygen species (ROS)^16–18^. In agreement with these previous findings, we found that TFAM knockdown in MKN45 cells increased both Ca^2+^ (Supplementary Figure S2C) and ROS levels (Supplementary Figure S2D), as determined by Fluo-4 AM and ROS-star 650 staining assays, respectively.

We then examined whether CFAP65 and PCK1 levels were dependent on the intracellular Ca^2+^ or ROS level. First, 60-min treatment with a selective Ca^2+^ chelator, 1,2-bis(o-aminophenoxy)ethane-*N*,*N*,*N*′,*N*′-tetraacetic acid (BAPTA), or 24-h treatment with an ROS inhibitor, *N*-acetylcysteine (NAC), was sufficient to decrease the intracellular Ca^2+^ (Supplementary Fig. S2C) and ROS levels (Supplementary Fig. S2D), respectively, in TFAM-knockdown MKN45 cells.

**Figure 5:**
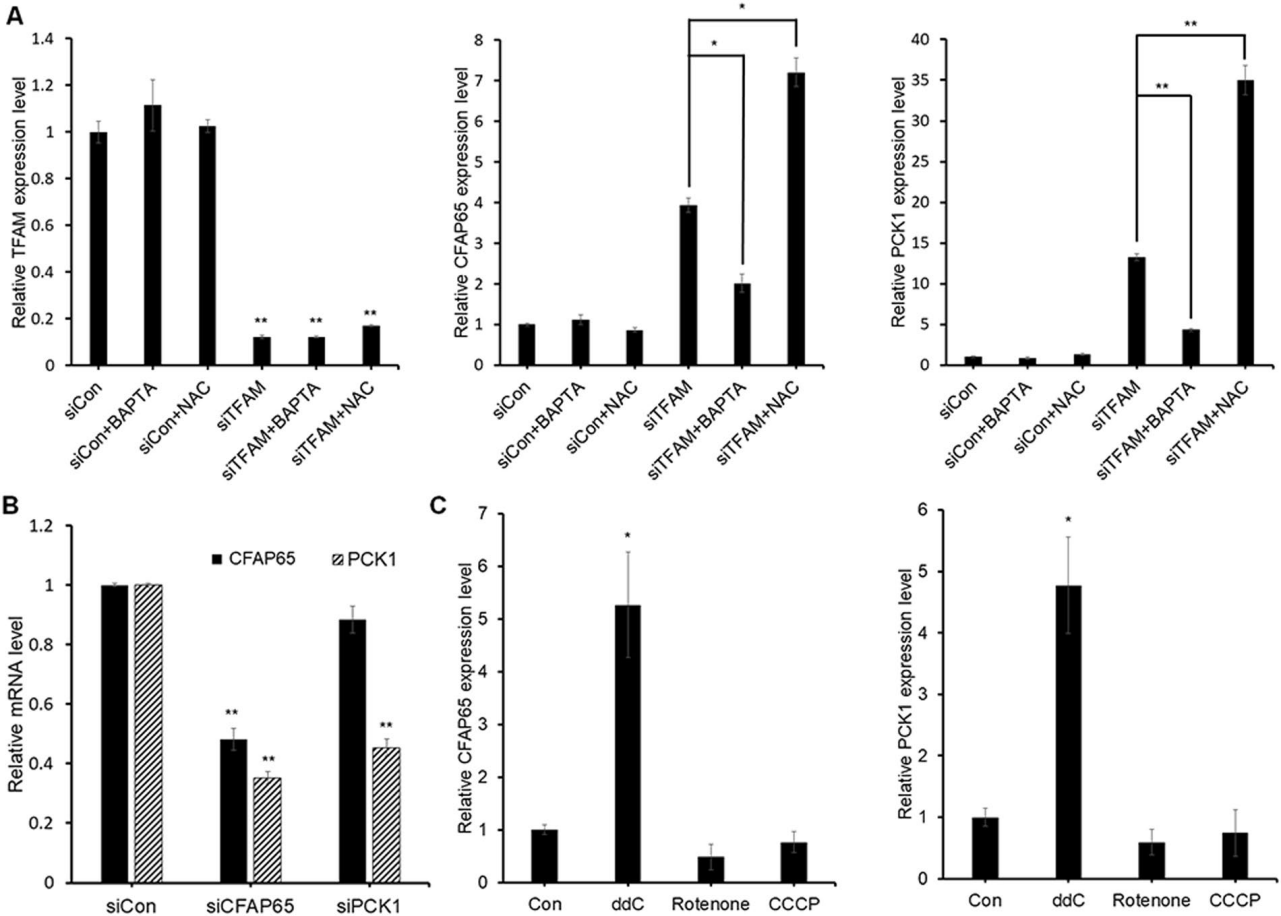
Effects of Ca^2+^- or ROS-mediated mitochondrial retrograde signaling on CFAP65 and PCK1. **(A)** TFAM, CFAP65, and PCK1 mRNA levels measured with or without 1-h treatment of 50 µM BAPTA or 24-h treatment of 10 mM NAC at 24 h after transfection using qPCR against GAPDH mRNA (*n* = 3, mean ± SD). Student’s t-test, *P < 0.05, **P < 0.01. **(B)** CFAP65 and PCK1 mRNA levels in siCFAP65- or siPCK1-transfected MKN45 cells (*n* = 3, mean ± SD). Student’s t-test, **P < 0.01. **(C)** Effects of ddC, rotenone, or CCCP treatment on CFAP65 and PCK1 mRNA levels in MKN45 cells measured with or without 48-h treatment of 200 µM ddC, 100 nM rotenone, or 10 µM CCCP (*n* = 3, mean ± SD). Student’s t-test, *P < 0.05.

Next, chelation of Ca^2+^ in TFAM-knockdown MKN45 cells decreased the mRNAs of both CFAP65 and PCK1, thus rescuing the effect of TFAM knockdown alone (Fig. 5A). By contrast, the inhibition of ROS increased rather than decreased the mRNAs of CFAP65, and did not rescue the TFAM knockdown effect. Together, these results indicate that Ca^2+^ -dependent, potentially calcineurin-mediated, mitochondrial retrograde signaling may participate in the TFAM knockdown-induced expression of *CFAP65* and *PCK1*.

### CFAP65 acting upstream of PCK1

To determine the relative order of CFAP65 and PCK1 involved in the TFAM-mediated transcription regulatory pathway, these genes were separately knocked down using siRNAs against each of them without TFAM depletion, after which the mRNAs of the two genes were quantified using qPCR (Fig. 5B). When CFAP65 was knocked down, PCK1 mRNA decreased in MKN45 cells. By contrast, PCK1 knockdown did not affect CFAP65 mRNA. These results suggested that CFAP65 acts upstream of PCK1 in the TFAM-mediated transcription regulatory pathway.

### Effects of mtDNA depletion on *CFAP65* and *PCK1* expression

TFAM knockdown decreased mitochondrial respiration and MMP of MKN45 cells (Supplementary Fig. S2B), consistent with previous findings^5,19^, so we checked whether disruption of oxidative phosphorylation or MMP can lead to the same gene expression changes observed with TFAM knockdown.

Rotenone inhibits mitochondrial complex I, and carbonyl cyanide m-chlorophenyl hydrazone (CCCP) dissipates MMP^20,21^. We also treated cells with 2′,3′-dideoxycytidine (ddC), which causes mtDNA depletion by inhibiting replication^22^. Treatment of ddC, rotenone and CCCP commonly decreased proliferation and increased cytoplasmic Ca^2+^ level of MKN45 cells (Supplementary Fig. S3) like TFAM knockdown, consistent with previous findings^23–25^. We also observed reduced MitoTracker staining, indicating the MMP reduction.

However, CFAP65 and PCK1 transcript levels were increased only by ddC treatment, but not by rotenone or CCCP treatment (Fig. 5C), suggesting that oxidative phosphorylation blockade or MMP depolarization is not sufficient for the induction of *CFAP65* and *PCK1* expression. Because mtDNA was depleted by either TFAM knockdown or ddC treatment but not by rotenone or CCCP treatment (Supplementary Fig. S3B), our results suggest that mtDNA depletion may be a critical factor to increase *CFAP65* and *PCK1* expression.

**Figure 6:**
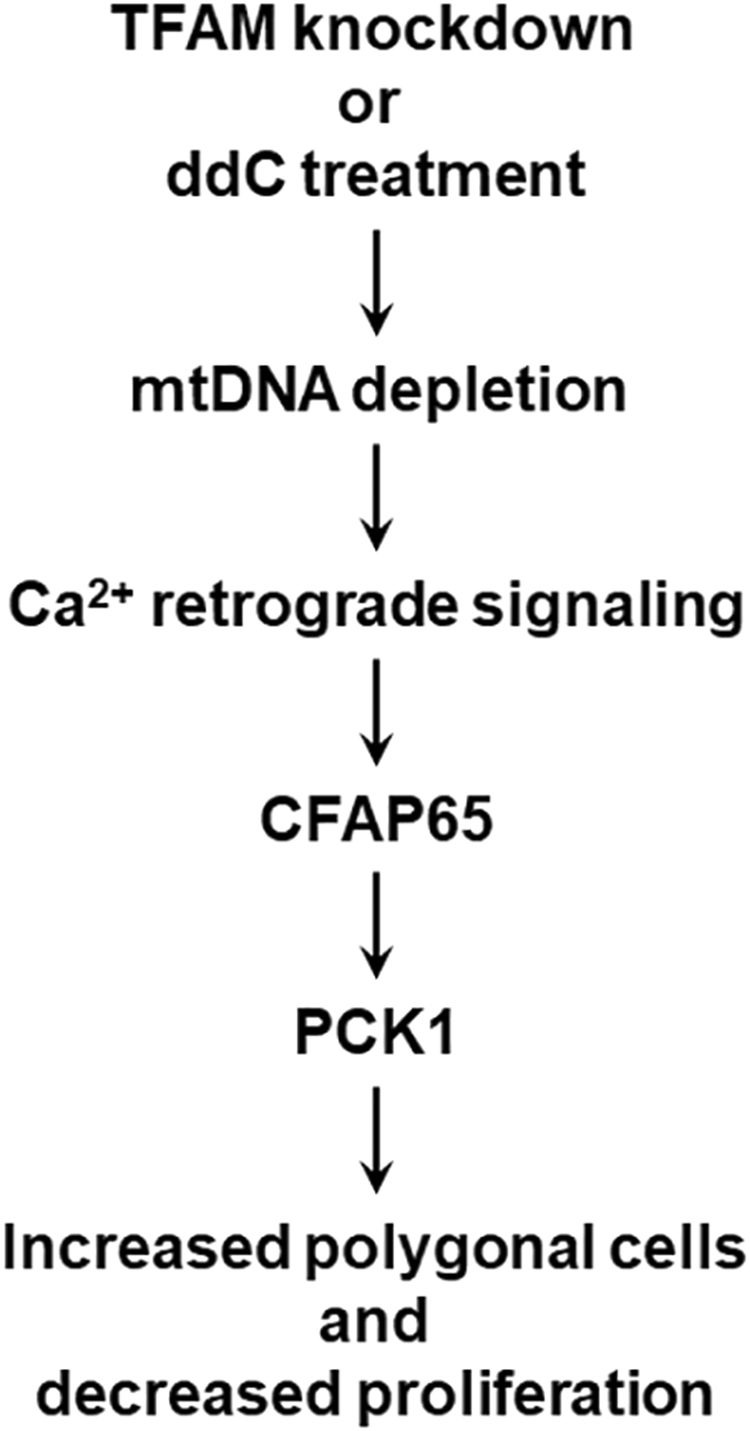
TFAM pathway of mtDNA–Ca^2+^ signal–CFAP65–PCK1 axis responsible for the proliferation and differentiation of MKN45 cells.

In summary, all the results together indicate that the TFAM–mtDNA–calcium–CFAP65–PCK1 axis participates in the mitochondrial retrograde signaling, affecting tumor cell differentiation and proliferation (Fig. 6). Specifically, TFAM knockdown, like ddC treatment, sequentially causes mtDNA depletion, calcium-mediated mitochondrial retrograde signaling, CFAP65 induction, and PCK1 induction, resulting in polygonalization and hypoproliferation of tumor cells.

### Association of *TFAM* with cancer risk

In a case-control disease-association study, 2,147 Korean participants including 941 gastric cancer patients and 1,206 healthy controls were genotyped for nine single-nucleotide polymorphisms (SNPs). These tag SNPs were selected within and around the *TFAM* gene, with a correlation coefficient *r* ^2^ ≥ 0.8, using the Asian haplotype data of the 1000 Genomes Project^26^.

Susceptibility to gastric cancer was not associated with any of these SNPs in an additive genetic model; however, in a dominant genetic model, susceptibility to diffuse-type gastric cancer (*n* = 393) was marginally associated with rs10826175 (*A* > *G*) SNP located in an upstream region (*GG* + *GA* versus *AA*, OR = 1.4, *P* = 0.019) and rs1937 (*G* > *C*) SNP located in exon 1 (*CC* + *GC* versus *GG*, OR = 1.3, *P* = 0.044) of *TFAM* after correction for multiple hypothesis testing (Table 2).

**Table 2:** Association of *TFAM* SNPs with gastric cancer risk.

SNP *B* > *b* ^a^	Controls^b^	All cases^c^	Diffuse-type gastric cancer^d^		
	MAF/*BB*/*Bb*/*bb*	MAF/*BB*/*Bb*/*bb*	MAF/*BB*/*Bb*/*bb*	OR (95% CI)	*P*
rs10826175 *A* > *G*	0.48/335/576/289	0.48/236/492/199	0.50/87/220/83	1.4 (1.1–1.9)	0.019
rs1937 *G* > *C*	0.18/813/349/44	0.19/610/309/22	0.20/245/137/11	1.3 (1.0–1.7)	0.044
rs2279339 *G* > *T*	0.18/809/343/44	0.19/614/296/29	0.20/247/132/14	1.3 (1.0–1.6)	0.054
rs16912196 *A* > *G*	0.30/582/510/104	0.30/463/390/80	0.29/199/162/31	0.9 (0.7–1.2)	0.49
rs117959541 *G* > *A*	0.19/789/371/46	0.20/598/309/33	0.20/248/134/11	1.1 (0.8–1.4)	0.55
rs2277256 *A* > *G*	0.058/1070/131/5	0.055/841/96/4	0.066/343/48/2	1.1 (0.8–1.6)	0.58
rs7086017 *G* > *A*	0.084/1008/188/7	0.083/791/143/7	0.085/330/59/4	0.9 (0.7–1.3)	0.68
rs7905675 *G* > *A*	0.50/296/616/292	0.49/238/482/221	0.50/103/189/101	1.0 (0.7–1.2)	0.71
rs11006131 *C* > *T*	0.065/1051/149/4	0.074/805/131/4	0.068/341/49/2	1.0 (0.7–1.5)	0.89

^a^SNPs are listed in ascending order of *P* values in the rightmost column. The major alleles are referred to as *B*, and the minor alleles as *b*.

^b^The genotypes of each SNP were in Hardy-Weinberg equilibrium (0.41 ≤ *P* ≤ 0.96) among the control subjects (*n* = 1,206).

^c^SNPs were not significantly associated with susceptibility to gastric cancer in additive genetic models (0.40 ≤ *P* ≤ 0.94) in comparison of all gastric cancer cases (*n* = 941) with the controls using logistic regression with adjustment for age, gender, and site of recruitment.

^d^The odds ratio (OR) and 95% confidence interval (CI) were calculated for the association of each SNP with susceptibility to diffuse-type gastric cancer (*n* = 393) in dominant genetic models using logistic regression with adjustment for age, gender, and site of recruitment.

Gene expression levels are highly heritable^27^, and their variations can be mapped to expression quantitative trait loci^28,29^. According to the data from the Genotype-Tissue Expression Project^30^, the cancer risk allele (*G*) of rs10826175 was associated with increased TFAM mRNA in various tissues, including the stomach and esophagus. These results suggested the association of increased *TFAM* expression with increased cancer susceptibility.

The rs1937 SNP, encoding a nonsynonymous change of serine-12 to threonine in the mitochondrial targeting peptide of TFAM, has been associated with susceptibility to late-onset and sporadic Alzheimer’s disease^31,32^. The other SNP, rs10826175, has been associated with susceptibility to coronary artery disease^33^, and its risk allele is associated with increased *TFAM* expression in many human tissues, including stomach tissue^30^.

Furthermore, according to the ONCOMINE microarray data, TFAM is upregulated in several cancer tissues, including gastric cancer tissues. Therefore, it is likely that the upregulation of TFAM is associated with increased cancer susceptibility.

## Discussion

The maintenance of cell polarity and integrity of the architecture is crucial for normal cell physiology and tissue homeostasis. In contrast, the loss of cell polarity and excessive cell growth are hallmarks of cancer^34^. The MKN45 cell line was established from a poorly differentiated adenocarcinoma that was metastatic to the liver^35^. MKN45 exhibits the loss of cell polarity, as evident in irregular arrangements and poorly defined margins of growing cells^36^, in agreement with our findings in this study, in which TFAM depletion in MKN45 cells transforming oval-shaped cells into growth-arrested polygonal cells. TFAM depletion-induced changes in cell morphology have not previously been reported, whereas those in proliferation inhibition have been reported in some cancer cells^8,37^.

*CFAP65* encoding cilia and flagella associated protein 65, also known as CCDC108 (coiled-coil domain containing 108), is stably expressed in differentiated cells, including human pluripotent stem cells^38^ and differentiated postnatal hair cells^39^. The MKN45 cell line possesses multi-potentiality for differentiation and produces a large population of CD44-positive cancer stem cells^40^. In this study, the upregulation of CFAP65 by TFAM depletion increased growth-arrested polygonal cells, thus implying that CFAP65 may have a novel function in driving cellular differentiation, because more differentiated cells proliferate less in general.

PCK1 refers to the cytoplasmic rather than the mitochondrial isoform of phosphoenolpyruvate carboxykinase (PEPCK-C), which catalyzes the cytoplasmic conversion of oxaloacetate into phosphoenolpyruvate, the rate-limiting, irreversible step of gluconeogenesis. mTORC2-dependent upregulation of PCK1 can be detrimental to cancer cell survival through the activation of gluconeogenesis^41^, whereas TP53-dependent downregulation of PCK1 may be relevant to tumor suppression in HEPG2 and HCT116 cells^42^.

Increased gluconeogenesis is associated with decreased tumorigenesis. Steroid-induced enhancement of gluconeogenesis decreases the formation of liver tumors in a mouse model^43^. The depletion of fructose-1,6-bisphosphatase, a regulator of gluconeogenesis, induces epithelial-mesenchymal transition (EMT) in breast cancer^44^ and is correlated with advanced tumor stage and poor prognosis in renal cell carcinoma^45^.

These previous findings are consistent with our data indicating that augmenting gluconeogenesis, probably by upregulation of PCK1, deleteriously affects cancer cell proliferation, although it remains to be investigated whether gluconeogenesis is involved in the tumor suppressor activity of PCK1.

TFAM knockdown results in mitochondrial dysfunction through the loss of mtDNA^13^ and the mitochondrial membrane potential^5^, thus evoking Ca^2+^-dependent mitochondrial retrograde signaling and subsequently increasing CFAP65 and PCK1 as observed in this study. Ca^2+^ mediated transcription factors that bind to the promoter of the *CFAP65* may increase the expression of *CFAP65*. *PCK1* expression has been reported to be regulated by the Ca^2+^-mediated CAMKII-FoxO1 pathway^46^.

However, we cannot rule out the possibility that a TFAM isoform that has recently been inferred to be imported into the nucleus^10,47^, may directly regulate the expression of *CFAP65* and *PCK1* as a nuclear transcription factor. Therefore, it remains to be investigated how Ca^2+^ signaling regulates *CFAP65* and *PCK1* expression.

Depletion of mtDNA is associated with glucose uptake reduction by decreased expression^48^ and translocation^49^ of glucose transporters. Furthermore, glucose deprivation increases PCK1 in human BJAB B-lymphoma cells^50^. Thus, we speculate that PCK1 is increased to promote gluconeogenesis in order to make up the shortage of glucose uptake caused by mtDNA depletion.

Inhibition of oxidative phosphorylation and reduction of membrane potential did not affect *CFAP65* and *PCK1* expression in this study, and conflicting study results were recently reported on TFAM depletion effects on glycolysis. TFAM deficiency sustains aerobic glycolysis in fibroblasts^19^, but inhibits glycolysis in non-small cell lung cancer^5^. Thus, we speculate TFAM depletion and other mitochondrial dysfunction may induce different metabolic programs depending on cancer types.

Increased expression of *TFAM* is a negative prognostic factor for survival among patients with tumor metastasis in colorectal cancer^51,52^, endometrioid cancer^53^, pancreatic cancer^4,54^, and ovarian cancer^55^. In addition, mitochondrial biogenesis and metabolism are required for anchorage-independent survival^56^ and tumor metastasis^57^. Overproduction of PGC-1α (peroxisome-proliferator-activated receptor coactivator-1α), which is a transcription activator of *TFAM* ^58^, promotes cell invasion and lung metastasis^59^.

These observations can be explained by migratory cancer cells requiring extra ATP and superoxide from mitochondrial biogenesis to survive under conditions imposed by metastatic colonization^59,60^. Our study results, together with previous findings, indicate that enhanced mitochondrial activity due to excessive TFAM may be associated with mitochondrial biogenesis-dependent tumor metastasis and poor patient survival. It remains to be investigated, however, whether Ca^2+^-dependent retrograde signaling and the CFAP65-PCK1 pathway is involved in mitochondrial biogenesis-dependent tumor metastasis.

TFAM depletion induces EMT when the mtDNA copy number is decreased by using ethidium bromide treatment or *TFAM* silencing in MCF7 cells^61^. By contrast, TFAM depletion has been found to induce the reverse transition—i.e., mesenchymal-epithelial transition—rather than EMT with decreased trans-well migration activity in an esophageal squamous cell carcinoma cell line^8^.

Likewise, it is possible that TFAM depletion in the MKN45 cell line leads to mesenchymal-epithelial transition, which converts motile oval-shaped cells to polarized polygonal cells. The conflicting results may have been caused by different efficiencies of TFAM or mtDNA depletion or by distinct characteristics of cancer cell lines.

In conclusion, TFAM depletion in MKN45 cells inhibits tumor progression, most probably by inducing differentiation into growth-arrested polygonal cells. This differentiation is promoted by the upregulation of CFAP65 and PCK1, which participate in the Ca^2+^-mediated mitochondrial retrograde signaling pathway. Furthermore, depletion of mtDNA is sufficient for upregulation of CFAP65 and PCK1 and downregulation of cell proliferation, whereas disruption of oxidative phosphorylation or MMP is not. Accordingly, mtDNA depletion would be a more effective therapeutic approach against TFAM-associated cancer.

## Methods

### Cell lines and cultures

The human gastric cancer cell line MKN45 was obtained from the Korean Cell Line Bank (Seoul, Korea). MKN45 cells were maintained in Gibco® RPMI-1640 medium (Life Technologies, Langley, OK) supplemented with 10% HyClone™ fetal bovine serum (Thermo Fisher Scientific, Waltham, MA) and 1% penicillin and streptomycin in a 5% CO_2_ atmosphere at 37 °C. Cells were kept in culture for four or fewer passages, and cell phenotypes were verified in every experiment.

### RNA interference

All siRNAs were purchased from Invitrogen to separately knock down TFAM (HSS144250 and HSS144251), BAK1 (s1880), CFAP65 (HSS151650), EFCAB12 (HSS131923), GLRX5 (s27700), ITPK1 (HSS105601), MRPS33 (HSS182165), NCAPG2 (s29694), NUPR1 (HSS123469), PCK1 (HSS107654), and RAN (HSS131923) and included the negative control scrambled siRNA med GC (12935–300). Cells were transfected with 20 nM control or anti-target siRNA using lipofectamine RNAimax reagent (Invitrogen, Carlsbad, CA) with 1:3 ratio of siRNA (μg) to lipofectamine (μl).

### Cell imaging

Time-lapse photography of live cell cultures with bright-field images were taken in a humidified chamber at 37 °C and 5% CO_2_ with a JuLI Stage real-time cell-history recorder (NanoEnTek, Seoul, Korea). Three-color confocal fluorescence microscopy for green, blue, and red was performed using an LSM 780 confocal laser scanning microscope (Carl Zeiss, Oberkochen, Germany).

### Cell proliferation assay

The 3-(4,5-dimethylthiazol-2-yl)-2,5-diphenyltetrazolium bromide (MTT) assay was performed using PrestoBlue® Cell Viability Reagent (Invitrogen). Eight thousand cells were grown in a 96-well plate for 6 days. MTT was added directly to the culture medium, and the cells were incubated at 37 °C for 30 min. The absorbance was read at 570 nm using a Fluoroskan Ascent™ microplate fluorometer (Thermo Fisher Scientific). A blank reference was taken from the control wells lacking cells. Data were obtained from three independent experiments in quintuplicate.

### Quantification of cell morphology changes

Image-Pro Plus software (Media Cybernetics, Silver Springs, MD) was used to count cells from the time-lapse images that were taken with the JuLI Stage and converted into 16-bit grayscale images. Total objects with select values of intensity (≤24,000) and area (10–520) in the recorded images were counted as cells using the ‘count and measure objects’ function. Among them, cell objects with a roundness ≥1.3 were regarded as polygonal, and those with a roundness <1.3 were regarded as oval-shaped.

### Western blotting and antibodies

Whole-cell lysates were prepared using Passive Lysis Buffer purchased from Promega (Madison, WI). The cell extracts (50 μg) were loaded on a 15% sodium dodecyl sulfate-polyacrylamide gel for electrophoresis, and proteins were transferred to polyvinylidene fluoride membranes with a Mini Trans-Blot® tank (Bio-Rad, Hercules, CA). The membrane was probed with antibodies against TFAM (#7019, Cell Signaling, Danvers, MA) and α-tubulin (CP06–100UG, Merck Millipore, Darmstadt, Germany). All secondary antibodies (Santa Cruz Biotechnology, Dallas, TX) were incubated for 1 h. Detection was performed using an Amersham ECL GST Western Blotting Detection Kit (GE Healthcare Life Sciences, Piscataway, NJ).

### RNA purification and quantification

Total RNA was isolated from cells with an RNA-spin™ Total RNA Extraction Kit purchased from iNtRON Biotechnology (Seongnam, Korea). cDNA was synthesized from oligo-dT primers with the ImProm-II™ Reverse Transcription System (Promega). qPCR was performed with the SYBR® Green Master Mix (Bio-Rad) and was analyzed with a CFX96 Touch™ Real-Time PCR Detection System (Bio-Rad). For primer sequences see Supplementary Table S2.

### RNA sequencing

An RNA library was prepared using a TruSeq Stranded Total RNA Library Prep Kit purchased from Illumina (San Diego, CA). Briefly, rRNA was removed from total RNA (700 ng) using an rRNA removal kit, and then RNA was cleaned up using RNA purification beads and fragmented with fragment mix at 94 °C for 6 min. Fragmented RNAs were primed with random hexamers and were reverse transcribed to first-strand cDNAs using reverse transcriptase and random primers in a PCR cycle of 25 °C for 10 min, 42 °C for 15 min, and 70 °C for 15 min. The replacement strands were then synthesized with dUTP instead of TTP to generate double-stranded cDNAs at 16 °C for 1 h. After the cDNAs were cleaned up using sample purification beads, ATP was added to extend the 3′ ends with an A-tailing mix reagent by incubating at 37 °C for 30 min, and then at 70 °C for 5 min.

Indexing adapters were ligated to the ends of the DNA fragments with ligation mix 2 reagent at 30 °C for 10 min. After washing twice with sample purification beads, PCR was carried out to enrich those DNA fragments that had adapter molecules on both ends. The thermocycler conditions were as follows: 95 °C for 3 min, 8 cycles of 98 °C for 20 s, 60 °C for 15 s, and 72 °C for 30 min, with a final extension at 72 °C for 5 min. Finally, the quality and band size of the library were assessed with an Agilent 2100 Bioanalyzer (Agilent, Santa Clara, CA). The libraries were quantified by qPCR with a CFX96 Touch™ Real-Time PCR Detection System (Bio-Rad). After normalization, sequencing of the prepared library was conducted with a NextSeq 500 System (Illumina).

### Quantitative transcriptome analysis

From each RNA-Seq library, an average of 55 million paired-end reads was generated. An RNA-Seq library was created as an overlapped library, and FastQC (v 0.11.5) was used for quality control of the library^62^. Trimmomatic software (v 0.33) was used to trim the Illumina universal sequencing adapter^63^. Reads less than 50 bp were discarded. TopHat software (v 2.0.13) was used for sequence alignment^64^. The reference sequence was hg19 from the UCSC genome browser (http://genome.ucsc.edu/). We calculated fragments per kilobase of exon per million fragments mapped (FPKM) using Cufflinks (v 2.2.1) and Cuffdiff software (v 2.2.1)^65^. For gene annotation, RefSeq and GENCODE (v 19) were used^66^.

### Quantification of the mtDNA copy number

Genomic DNA was purified with a QlAamp® DNA Mini Kit (Qiagen GmbH, Hilden, Germany). qPCR was performed with a CFX96 Touch™ Real-Time PCR Detection System (Bio-Rad). mtDNA copy number was estimated using ∆Cq of mtDNA(ND1)/nDNA(18 S rRNA), as previously described^8^. Mitochondria inhibitors, ddC (D5782), rotenone (R8875), and CCCP (C2759), were purchased from Sigma-Aldrich.

### Quantification of cellular Ca^2+^ and ROS levels

The cellular Ca^2+^ level was assessed by 30-min incubation of 5 µM Fluo-4 AM (Thermo Fisher Scientific), a fluorogenic marker for Ca^2+^ in live cells. After incubation and washing, fluorescence was analyzed using a JuLI Stage, with excitation at 494 nm and emission at 506 nm. The cellular ROS level was assessed after 30-min incubation of 25 µM ROSstar™ 650 (LI-COR Biosciences, Lincoln, NE), a hydrocyanine probe for ROS. After incubation, cells were washed with phosphate-buffered saline buffer, and fluorescence was analyzed with a JuLI Stage, with excitation at 638 nm and emission at 656 nm. Fluorescence signals were quantified using Image-Pro® Plus software (Media Cybernetics, Silver Springs, MD) and were expressed as the mean fluorescence intensity per cell.

### Study subjects and DNA genotyping

A total of 2,147 Korean participants were recruited at five university-affiliated hospitals, as described previously^67^, and additionally from Pusan National University Hospital, under approval from the Institutional Review Board of each university hospital. All individuals provided informed consent. The participants comprised 1,206 healthy controls and 941 patients with either diffuse-type (*n* = 393) or intestinal-type gastric cancer (*n* = 548). Genomic DNA samples were purified from the peripheral blood of the participants and were genotyped using a MassARRAY iPLEX platform (Sequenom, San Diego, CA) under approval from the Institutional Review Board of Korean Advanced Institute of Science and Technology. All experiments were performed in accordance with the approved guidelines. The call rate per SNP was 98% or higher, and all genotypes of the healthy controls were in Hardy-Weinberg equilibrium (*P ≥ *0.05). Their demographic characteristics are shown in Supplementary Table S3.

### Statistical analysis

All qPCR experiments were performed with three replicated samples each in triplicate. Data are expressed as the mean values ± standard error and were analyzed by Student’s t-test or Mann–Whitney test using SPSS 11.5 (SPSS, Chicago, IL). For genotyping analysis, the Haploview 4.1 program (Broad Institute, Cambridge, MA) was used for the selection of tag SNPs, calculation of pairwise linkage disequilibrium values between SNPs, and construction of a linkage disequilibrium map using the genotype data for the Asian populations of the International HapMap Project. SPSS 11.5 (SPSS, Chicago, IL) was used to calculate the odds ratio, 95% confidence interval, and *P*-value for each SNP in association tests of multivariate logistic regression analyses.

## Electronic supplementary material

Supplementary information

Supplementary Video S1
