# Supplementary material for: Transcriptomic analysis of mitochondrial TFAM depletion changing cell morphology and proliferation
Source: Sci Rep. 2017 Dec 19;7:17841. doi: 10.1038/s41598-017-18064-9 (PMC5736646; doi:10.1038/s41598-017-18064-9)
Supplement: Supplementary file 1 — Supplementary information [file 41598_2017_18064_MOESM1_ESM.doc]

**Supplementary Materials**

**Transcriptomic analysis of mitochondrial TFAM depletion changing cell morphology and proliferation**

Woo Rin Lee,^1,2^ Heeju Na,^1^ Seon Woo Lee,^1^ Won-Jun Lim,^3,4^ Namshin Kim,^3,4^ J. Eugene Lee,^2^ and Changwon Kang^1^

^1^ Department of Biological Sciences, Korea Advanced Institute of Science and Technology, Daejeon 34141, Korea

^2^ Center for Bioanalysis, Korea Research Institute of Standards and Science, Daejeon 34113, Korea

^3^ Personalized Genomic Medicine Research Center, Korea Research Institute of Bioscience and Biotechnology, Daejeon 34141, Korea

^4^ Department of Functional Genomics, KRIBB School of Bioscience, Korea University of Science and Technology, Daejeon 34141, Korea

Correspondence and requests for materials should be addressed to C.K. (email: ckang@kaist.ac.kr) or J.E.L. (email: j.eugenelee@gmail.com)

**List of supplementary materials**

Video S1. Effects of TFAM depletion on the morphology of MKN45 cells. [p. 1]

Figure S1. Full western blots of Fig. 1 in high and low contrast. [p. 2]

Figure S2. Effects of TFAM depletion on Ca^2+^- or ROS-mediated signaling. [p. 3]

Figure S3. Effects of ddC, rotenone, and CCCP on MKN45 cells. [p. 4]


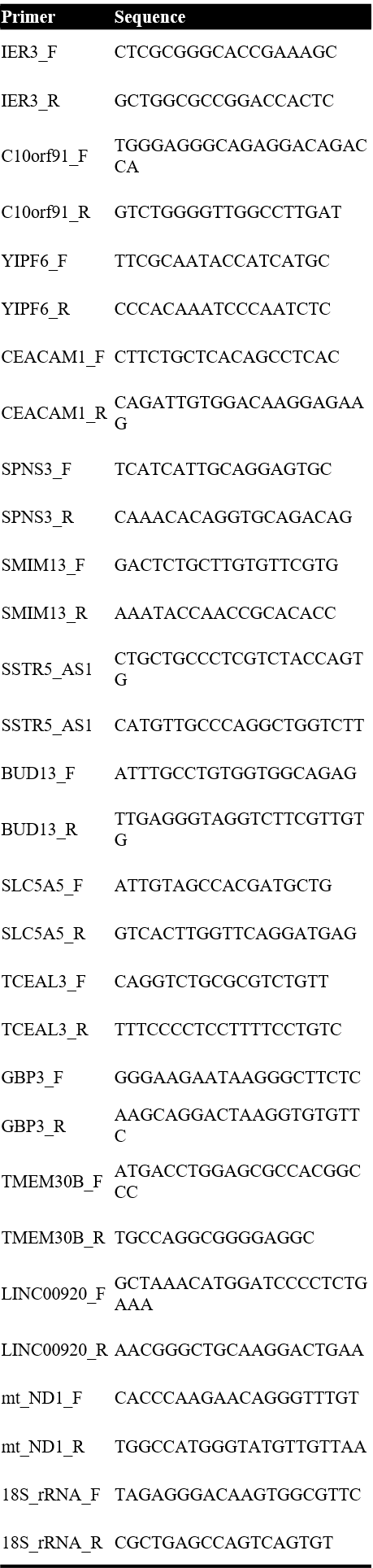
Table S1. List of 101 genes affected more than 2-fold in RNA-Seq by TFAM depletion. [pp. 5-6]


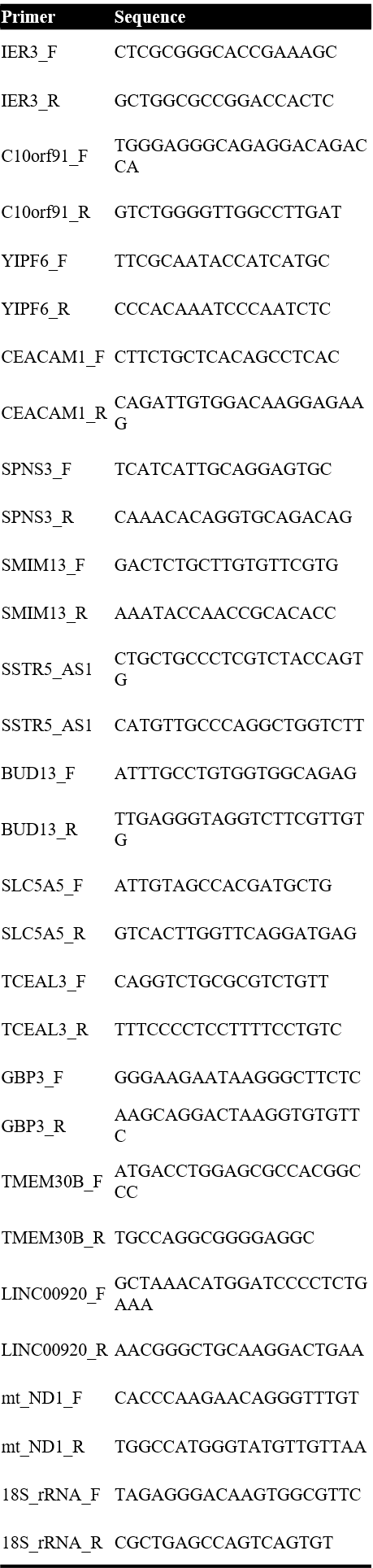
Table S2. List of 68 qPCR primer pairs. [pp. 7-9]


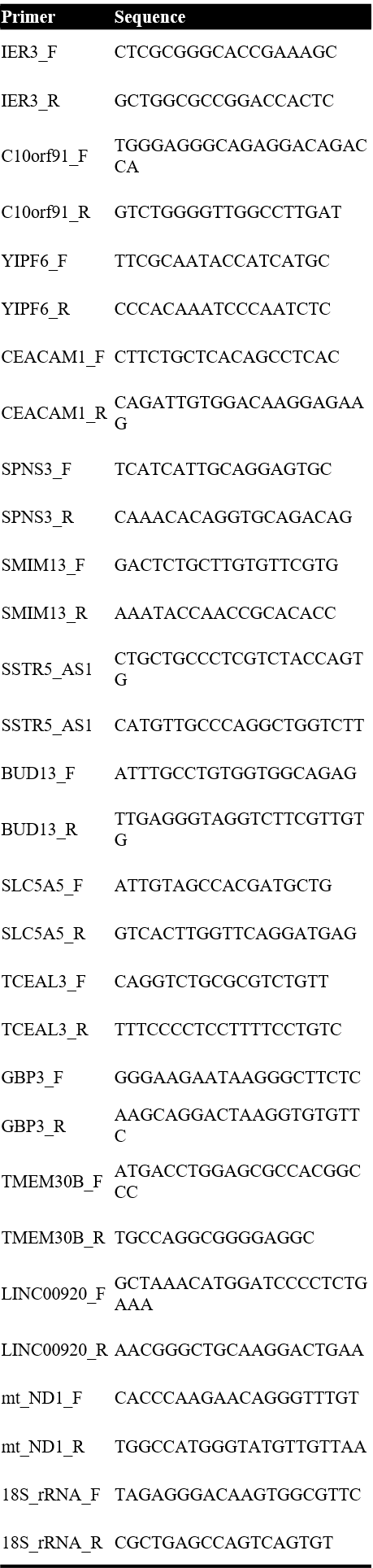
Table S3. Demographic characteristics for the SNP association study of Table 2. [p. 9]

**Supplementary Video S1.** Effects of TFAM depletion on the morphology of MKN45 cells.


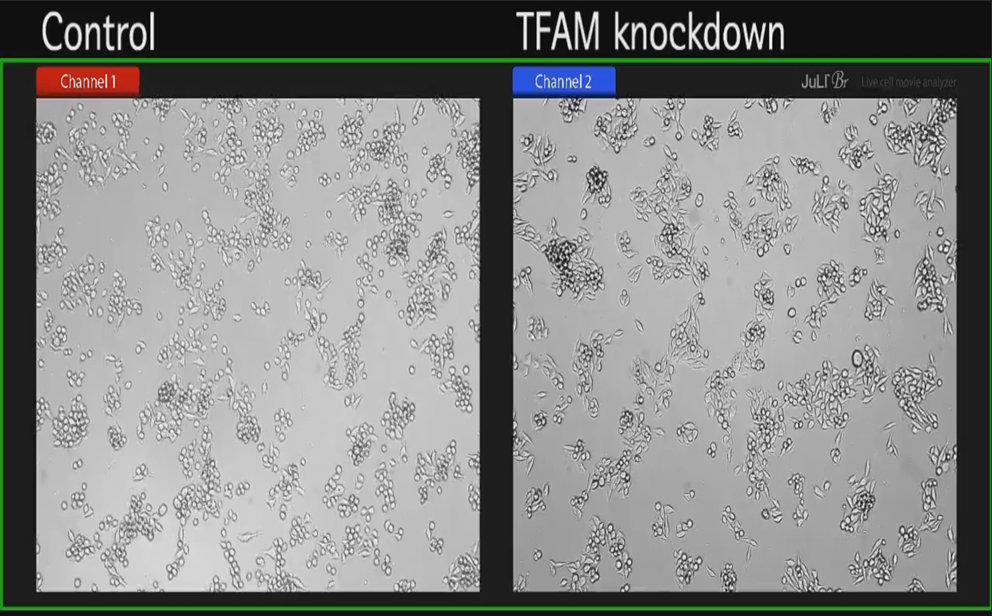


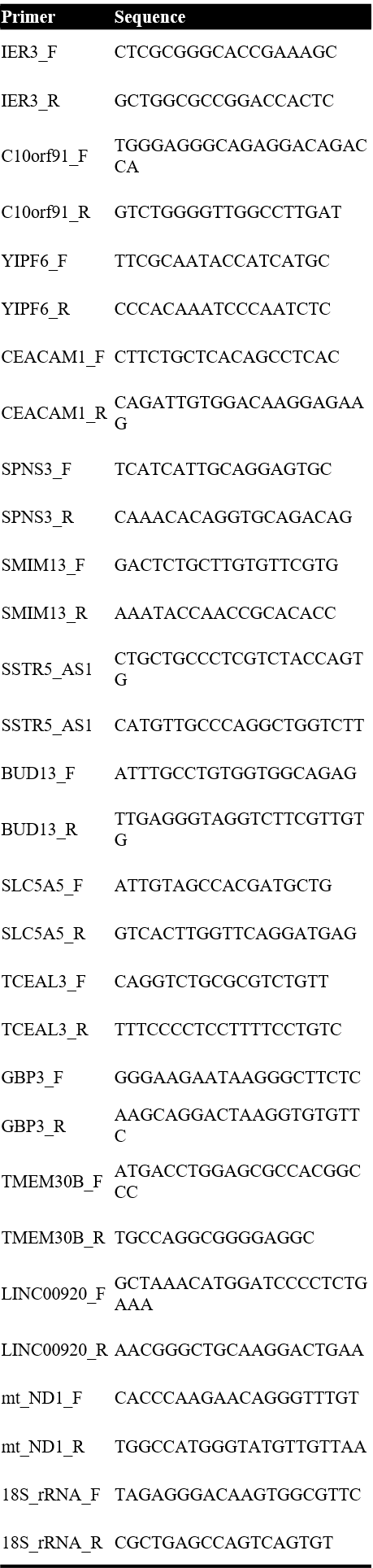
Live cell images were recorded for 96 h after transfection with siTFAM#1, but only the initial 54 h recording is included in this clip. Frames were collected every 10 min, and videos display at 19 frames per second.

**Supplementary Figure S1.** Full western blots of Fig. 1 in high and low contrast.

High contrast:

**
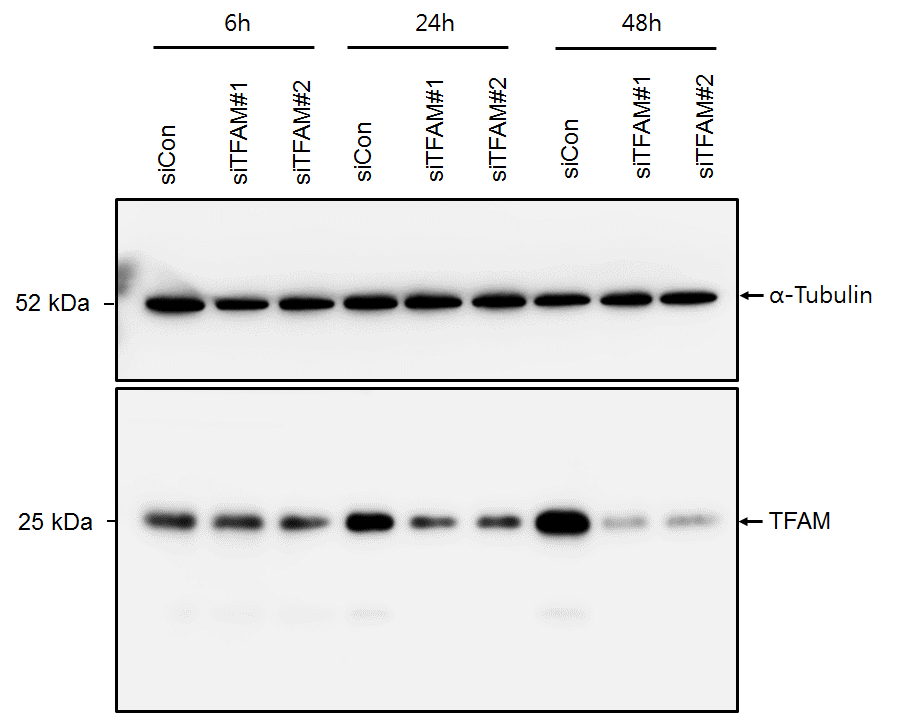
**

Low contrast:

**
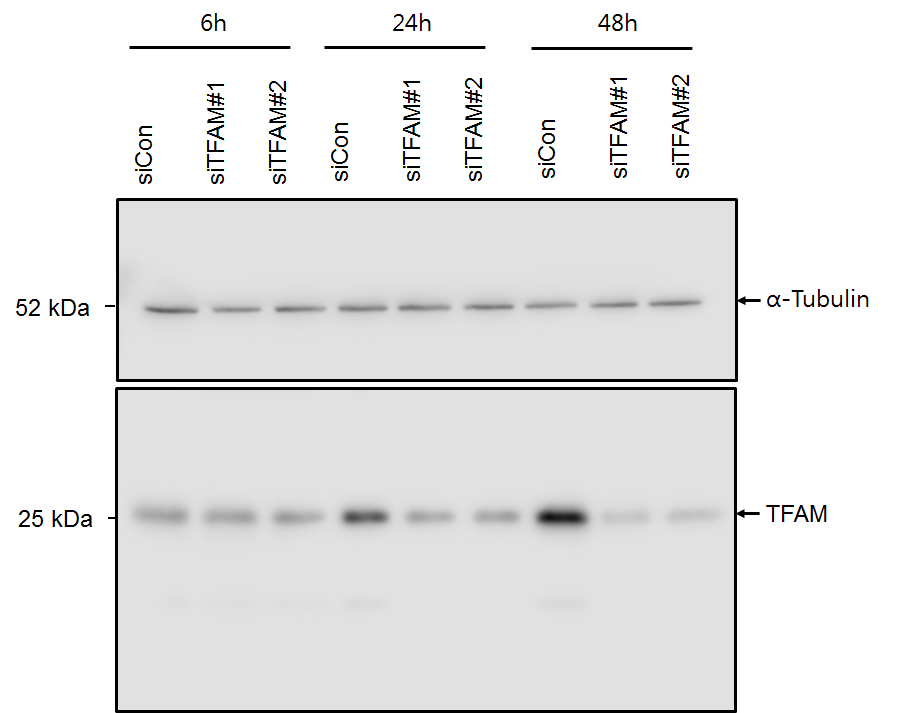
**

**Supplementary Figure S2.** Effects of TFAM depletion on Ca^2+^- or ROS-mediated signaling.


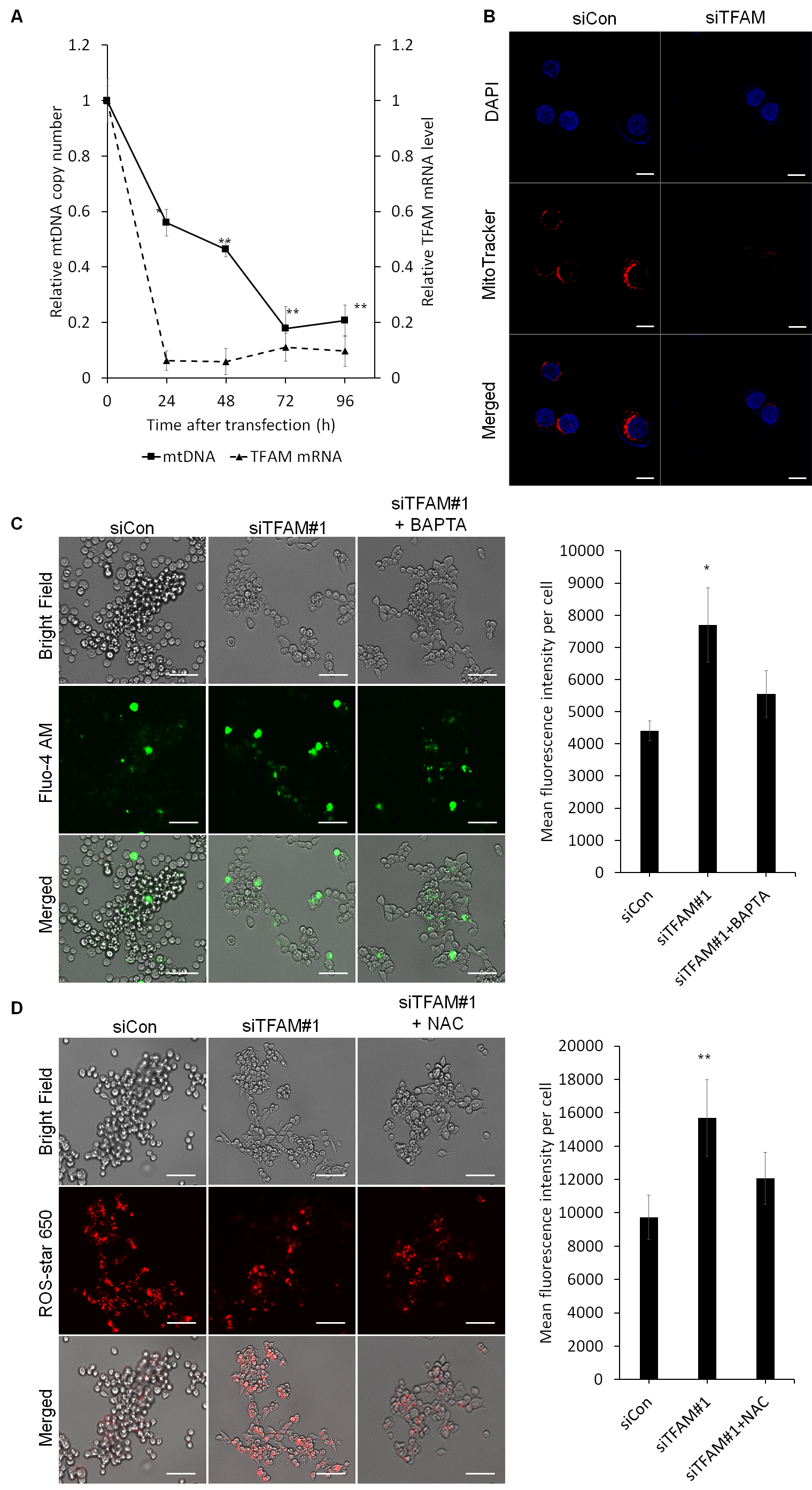


**(A)** The mtDNA copy number and TFAM mRNA level measured using qPCR (*n* = 3, mean ± SD). Student’s t-test * *P* < 0.05, ** *P* < 0.01. **(B)** Representative confocal images of DAPI- and MitoTracker-stained TFAM-knockdown MKN45 cells were taken at 48 h after transfection using LSM 780. The scale bar represents 10 µm. **(C)** Left panel: representative bright-field and immunofluorescence images of MKN45 cells treated with a Ca^2+^ chelator and siTFAM#1. The scale bar represents 50 µm. Right panel: mean fluorescence intensity per cell. * *P* < 0.05. **(D)** Left panel: representative bright-field and immunofluorescence images of MKN45 cells treated with an ROS chelator and siTFAM#1. The scale bar represents 50 µm. Right panel: mean fluorescence intensity per cell. ** *P* < 0.01.

**Supplementary Figure S3.** Effects of ddC, rotenone, and CCCP on MKN45 cells


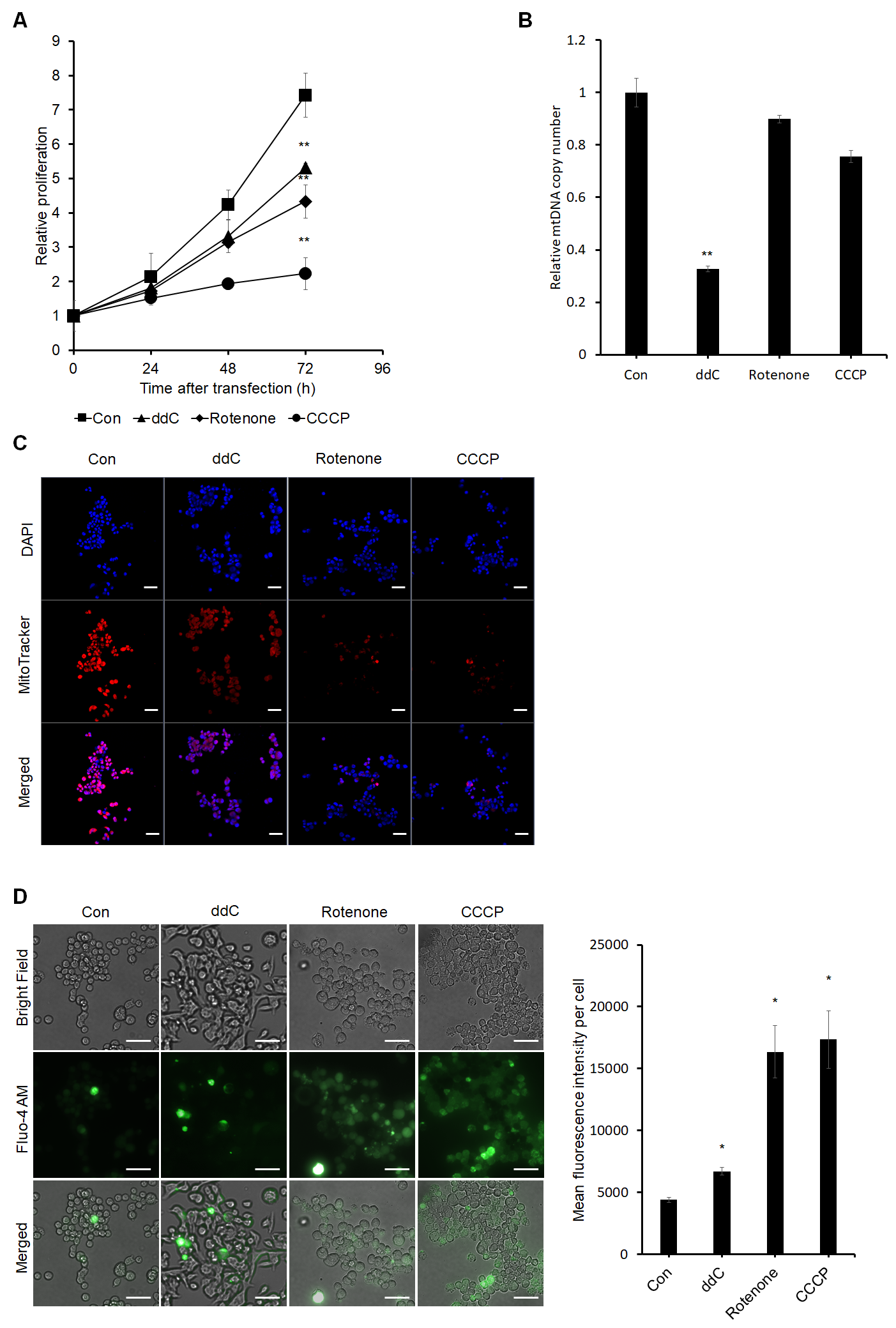


**(A)** MTT assay of MKN45 cells with or without treatment of 200 µM ddC, 100 nM rotenone, or 10 µM CCCP. *A*_570 nm_ was measured at 0, 24, 48, and 72 h after treatment (*n* = 3, mean ± SD). Student’s t test, ** *P* < 0.01. **(B)** The mtDNA copy number measured using qPCR (*n* = 3, mean ± SD). Student’s t-test ** *P* < 0.01. **(C)** Representative confocal images of DAPI- and MitoTracker-stained MKN45 cells with or without 48-h treatment of 200 µM ddC, 100 nM rotenone, or 10 µM CCCP were taken using LSM 880. The scale bar represents 50 µm. **(D)** Left panel: representative bright-field and immunofluorescence images of MKN45 cells with or without 48-h treatment of 200 µM ddC, 100 nM rotenone, or 10 µM CCCP. The scale bar represents 50 µm. Right panel: mean fluorescence intensity per cell. * *P* < 0.05.

**Supplementary**
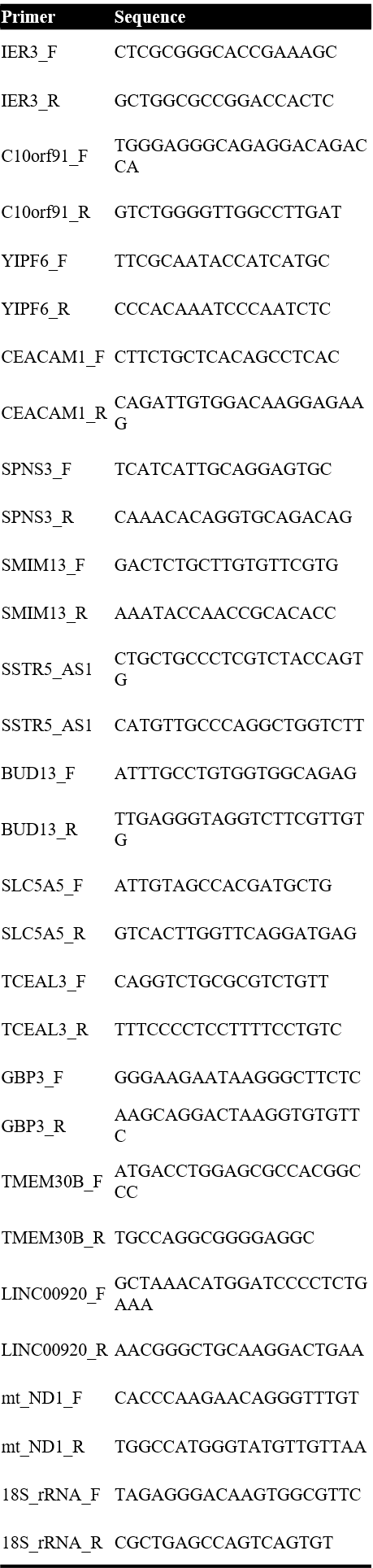
**Table S1.** List of 101 genes affected more than 2-fold in RNA-Seq by TFAM depletion. The genes are listed in descending order of the absolute values of fold changes (FC) by qPCR at 24 h after siRNA transfection. The fold changes were first semi-quantified using RNA-Seq data (Mann Whitney test, * *P* < 0.05) and next quantified using qPCR (*n* = 3, Student t test, * *P* < 0.05).

| **Rank** | **Gene** | **FC by qPCR** | | |  | **FC by RNA-Seq** | | |
| --- | --- | --- | --- | --- | --- | --- | --- | --- |
|  |  | **6 h** | **24 h** | **48 h** |  | **6 h** | **24 h** | **48 h** |
| 1 | *TFAM* | 0.22* | 0.13* | 0.16* |  | 0.27* | 0.11* | 0.19* |
| 2 | *NUPR1* | 1.1 | 6.9* | 16* |  | 0.85 | 2.5* | 13 |
| 3 | *ITPK1* | 1.1 | 0.17* | 0.23* |  | 0.88* | 0.48* | 0.31* |
| 4 | *EFCAB12* | 1.2 | 5.3* | 6.2* |  | 0.92 | 3.1* | 4.0 |
| 5 | *PCK1* | 1.1 | 4.5* | 12* |  | 1.2 | 3.1* | 6.9 |
| 6 | *CFAP65* | 1.5 | 4.5* | 6.1* |  | 0.92 | 2.0* | 3.3 |
| 7 | *GLRX5* | 1.1 | 0.23* | 0.21* |  | 0.89 | 0.41* | 0.32 |
| 8 | *UBLCP1* | 0.51* | 0.24* | 0.31* |  | 0.68* | 0.49* | 0.42 |
| 9 | *BAK1* | 0.87 | 0.32* | 0.31* |  | 0.66* | 0.36* | 0.35* |
| 10 | *MRPS33* | 0.93 | 0.32* | 0.31* |  | 0.86 | 0.23* | 0.33* |
| 11 | *NCAPG2* | 0.92 | 0.33* | 0.32* |  | 0.78* | 0.41* | 0.30* |
| 12 | *RAN* | 0.88 | 0.33* | 0.42* |  | 0.84 | 0.32* | 0.29 |
| 13 | *KRT80* | 0.45* | 0.34* | 0.28* |  | 0.67* | 0.48* | 0.46* |
| 14 | *GALNT1* | 1.0 | 0.35* | 0.21* |  | 0.87 | 0.45* | 0.35 |
| 15 | *CALHM3* | 1.1 | 0.35* | 0.23* |  | 0.89 | 0.43* | 0.22 |
| 16 | *MRPL33* | 0.6 | 0.35* | 0.35* |  | 1.1 | 0.38* | 0.59 |
| 17 | *PGM2* | 0.86 | 0.36* | 0.32* |  | 0.86 | 0.45* | 0.39 |
| 18 | *THRIL* | 0.92 | 0.37* | 0.39* |  | 0.85* | 0.48* | 0.41 |
| 19 | *NEK10* | 1.4 | 2.7* | 2.8* |  | 1.2 | 2.5* | 4.9* |
| 20 | *GTF3C6* | 1.1 | 0.39* | 0.55* |  | 0.98 | 0.44* | 0.40 |
| 21 | *GALNT3* | 0.94 | 0.41* | 0.43* |  | 0.76* | 0.46* | 0.44* |
| 22 | *SH3TC2* | 0.82 | 0.41* | 0.21* |  | 0.91 | 0.47* | 0.24 |
| 23 | *THRAP3* | 0.91 | 0.41* | 0.42* |  | 0.83* | 0.45* | 0.45 |
| 24 | *RAB31* | 0.92 | 0.42* | 0.32* |  | 0.82 | 0.47* | 0.31 |
| 25 | *AP1AR* | 0.60* | 0.42* | 0.27* |  | 0.77* | 0.39* | 0.43* |
| 26 | *DDX21* | 0.93 | 0.43* | 0.41* |  | 0.9 | 0.46* | 0.36 |
| 27 | *LIF* | 0.94 | 0.43* | 0.43* |  | 0.94 | 0.47* | 0.34 |
| 28 | *CERK* | 0.91 | 0.43* | 0.47* |  | 0.80* | 0.41* | 0.47* |
| 29 | *PAQR5* | 1.1 | 0.43* | 0.23* |  | 0.92 | 2.1* | 2.3 |
| 30 | *CHMP5* | 0.93 | 0.44* | 0.38* |  | 0.93 | 0.35* | 0.44* |
| 31 | *COPS8* | 0.84 | 0.44* | 0.40* |  | 0.95 | 0.48* | 0.52 |
| 32 | *CCDC65* | 1.1 | 2.3* | 3.1* |  | 1.2 | 2.0* | 3.1 |
| 33 | *HYAL1* | 0.91 | 0.45* | 0.40* |  | 0.89 | 0.45* | 0.48 |
| 34 | *LY6D* | 0.97 | 0.45* | 0.53* |  | 1.1 | 0.46* | 0.29 |
| 35 | *GRHPR* | 0.75 | 0.45* | 0.30* |  | 0.95 | 0.42* | 0.38 |
| 36 | *ANKRD13A* | 0.81* | 0.47* | 0.36* |  | 0.87 | 0.49* | 0.54* |
| 37 | *SLC9A2* | 0.97 | 0.48* | 0.51* |  | 0.70* | 0.43* | 0.40* |
| 38 | *SLK* | 0.8 | 0.48* | 0.32* |  | 0.70* | 0.48* | 0.52* |
| 39 | *EDEM1* | 0.92 | 0.49* | 0.43* |  | 0.62* | 0.46* | 0.36* |
| 40 | *SEC24D* | 0.6 | 0.50* | 0.62* |  | 0.75* | 0.49* | 0.49* |
| 41 | *CISD1* | 0.98 | 0.51* | 0.43* |  | 0.95 | 0.45* | 0.5 |
| 42 | *RNF38* | 1.1 | 1.9* | 4.2* |  | 1.2 | 2.2* | 2.4 |
| 43 | *PROCR* | 1.3 | 0.53* | 0.52* |  | 1.0 | 0.46* | 0.39 |
| 44 | *SLC26A9* | 1.1 | 0.53* | 0.51* |  | 0.88 | 0.47* | 0.18 |
| 45 | *SPDEF* | 1.3 | 0.53* | 0.51 |  | 0.91 | 0.45* | 0.35* |
| 46 | *LRG1* | 1.2* | 0.55* | 0.48* |  | 0.93 | 0.47* | 0.50* |
| 47 | *SUMO2* | 0.94 | 0.56 | 0.64 |  | 0.96 | 0.49* | 0.48 |
| 48 | *CCRL2* | 1.7 | 0.56* | 0.32* |  | 0.78 | 0.49* | 0.3 |
| 49 | *LMCD1-AS1* | 1.1 | 0.57* | 0.78 |  | 1.1 | 2.5* | 1.8 |
| 50 | *C6orf165* | 0.73 | 1.73 | 2.36 |  | 1.0 | 2.7* | 2.9 |
| 51 | *CENPB* | 0.89 | 0.58* | 0.73 |  | 0.89 | 0.45* | 0.34 |
| 52 | *CXCL8* | 1.1 | 0.59* | 0.33* |  | 1.1 | 0.48* | 0.27 |
| 53 | *TNNC1* | 0.85 | 0.62 | 0.44 |  | 1.1 | 0.49* | 0.42* |
| 54 | *IL6R* | 1.1 | 1.5* | 2.2* |  | 0.92 | 2.1* | 3.7 |
| 55 | *IER3* | 1.0 | 0.66* | 0.41* |  | 1.1 | 0.48* | 0.40 |
| 56 | *C10orf91* | 1.1 | 0.70* | 0.55* |  | 0.92 | 0.43* | 0.40* |
| 57 | *YIPF6* | 1.1 | 0.72* | 0.8 |  | 0.69* | 0.36* | 0.40* |
| 58 | *CEACAM1* | 0.97 | 0.74* | 0.78 |  | 0.75 | 0.49* | 0.30 |
| 59 | *SPNS3* | 0.94 | 0.74 | 2.2* |  | 1.1 | 0.48* | 0.52 |
| 60 | *SMIM13* | 1.0 | 0.75* | 0.72 |  | 0.77* | 0.45* | 0.41 |
| 61 | *SSTR5-AS1* | 1.3 | 1.29 | 1.66 |  | 1.1 | 2.5* | 1.1 |
| 62 | *BUD13* | 1.11 | 0.79* | 0.60* |  | 0.85 | 0.50* | 0.51 |
| 63 | *SLC5A5* | 0.97 | 0.82 | 0.8 |  | 0.96 | 2.4* | 3.4 |
| 64 | *TCEAL3* | 1.1 | 0.82 | 0.9 |  | 1.4 | 0.49* | 0.74 |
| 65 | *GBP3* | 1.2 | 0.87 | 1.1 |  | 0.85 | 0.49* | 0.75* |
| 66 | *TMEM30B* | 1.3* | 1.1 | 1.2* |  | 0.62* | 0.36* | 0.32* |
| 67 | *LINC00920* | 0.69 | 1.0 | 1.1 |  | 0.71* | 0.36* | 0.70 |
| 68 | *MIR4521* | N/A | N/A | N/A |  | 1.3 | 0.43* | 0.79 |
| 69 | XLOC_027016 | N/A | N/A | N/A |  | 2.1 | 4.2* | 4.1 |
| 70 | XLOC_003027 | N/A | N/A | N/A |  | 0.73 | 0.25* | 0.39 |
| 71 | XLOC_019016 | N/A | N/A | N/A |  | 1.5 | 0.25* | 0.67 |
| 72 | XLOC_031142 | N/A | N/A | N/A |  | 2.4* | 0.26* | 0.52 |
| 73 | XLOC_031086 | N/A | N/A | N/A |  | 2.3 | 0.26* | 1.9* |
| 74 | XLOC_031111 | N/A | N/A | N/A |  | 1.8 | 0.27* | 0.62 |
| 75 | XLOC_002787 | N/A | N/A | N/A |  | 1.1 | 0.27* | 0.45 |
| 76 | XLOC_016925 | N/A | N/A | N/A |  | 0.83 | 3.0* | 2.2 |
| 77 | XLOC_020538 | N/A | N/A | N/A |  | 1.1 | 0.34* | 1.2 |
| 78 | XLOC_030079 | N/A | N/A | N/A |  | 0.91 | 0.34* | 0.93 |
| 79 | XLOC_030072 | N/A | N/A | N/A |  | 1.8 | 0.36* | 1.1 |
| 80 | XLOC_024179 | N/A | N/A | N/A |  | 1.3 | 0.36* | 0.79 |
| 81 | XLOC_011793 | N/A | N/A | N/A |  | 1.3 | 2.7* | 8.8 |
| 82 | XLOC_031143 | N/A | N/A | N/A |  | 1.8 | 0.38* | 0.44 |
| 83 | XLOC_001599 | N/A | N/A | N/A |  | 1.9* | 0.40* | 0.72 |
| 84 | XLOC_031109 | N/A | N/A | N/A |  | 1.7 | 0.41* | 0.46 |
| 85 | XLOC_030123 | N/A | N/A | N/A |  | 1.0 | 0.41* | 1.2 |
| 86 | XLOC_017916 | N/A | N/A | N/A |  | 1.5 | 0.42* | 1.6 |
| 87 | XLOC_031105 | N/A | N/A | N/A |  | 1.7 | 0.42* | 1.4 |
| 88 | XLOC_012977 | N/A | N/A | N/A |  | 1.2 | 0.42* | 1.1 |
| 89 | XLOC_030087 | N/A | N/A | N/A |  | 1.4 | 0.42* | 0.94 |
| 90 | XLOC_013131 | N/A | N/A | N/A |  | 1.6 | 0.43* | 1.0 |
| 91 | XLOC_030125 | N/A | N/A | N/A |  | 1.1 | 0.44* | 0.31 |
| 92 | XLOC_026461 | N/A | N/A | N/A |  | 1.5 | 0.45* | 1.7 |
| 93 | XLOC_030126 | N/A | N/A | N/A |  | 1.3 | 0.45* | 0.47 |
| 94 | XLOC_031081 | N/A | N/A | N/A |  | 1.3 | 0.45* | 0.84 |
| 95 | XLOC_033336 | N/A | N/A | N/A |  | 1.2 | 2.2* | 2.2 |
| 96 | XLOC_008697 | N/A | N/A | N/A |  | 1.1 | 0.46* | 0.77 |
| 97 | XLOC_031097 | N/A | N/A | N/A |  | 1.4 | 0.46* | 0.90 |
| 98 | XLOC_021069 | N/A | N/A | N/A |  | 1.3 | 0.46* | 0.66 |
| 99 | XLOC_027934 | N/A | N/A | N/A |  | 0.92 | 2.1* | 0.93 |
| 100 | XLOC_030069 | N/A | N/A | N/A |  | 2.1* | 0.48* | 1.2 |
| 101 | XLOC_035380 | N/A | N/A | N/A |  | 1.0 | 2.0* | 2.8 |

**Supplementary**
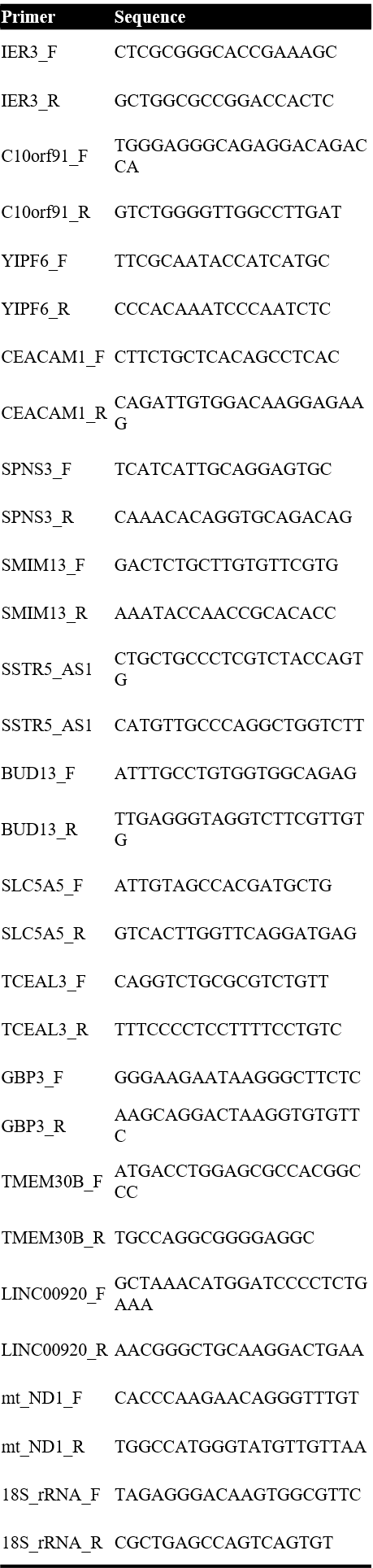
**Table S2.** List of 68 qPCR primer pairs.

| **Primer** | **Sequence** |
| --- | --- |
| TFAM_F | AGCTCAGAACCCAGATGC |
| TFAM_R | CCACTCCGCCCTATAAGC |
| NUPR1_F | AGACAGAGCTGGAGATGAGG |
| NUPR1_R | CGTCTCTATTGCTGGGTGTAG |
| ITPK1_F | GCCTACATGGAAGACGACAG |
| ITPK1_R | CAAATGAATGGGAAAGTCAAG |
| EFCAB12_F | TTGTCGCTGCTCGGACTC |
| EFCAB12_R | CCTTCTGCTGGAACTGCTTG |
| PCK1_F | GTATGACAACTGCTGGTTGG |
| PCK1_R | ACTGTGTCTCTTTGCTCTTGG |
| CCDC108_F | ATGGCCGGGAGATCCTGCTAA |
| CCDC108_R | CTGCCGTGGGGGTAGCG |
| GLRX5_F | GTGGTGGTCTTCCTCAAG |
| GLRX5_R | GTCTTTAATGCCTTGTCG |
| UBLCP1_F | CAAAGTTAAAGGCAAACCTG |
| UBLCP1_R | ATCTTCCAAGCTCTCCTCAC |
| BAK1_F | AGGTAGCCCAGGACACAG |
| BAK1_R | CATGGTGCTGCTAGGTTG |
| MRPS33_F | GGTATCCAAATCACCACACTTAC |
| MRPS33_R | TGATGCTCATCTCTGTAGAGTC |
| NCAPG2_F | AAATACTGGAGGCGATTG |
| NCAPG2_R | CTCCTCGGAAGGTGTATC |
| RAN_F | GAAGAAGTATGTAGCCACCTTG |
| RAN_R | AATAGGTCCTCTGTTGGTGTG |
| KRT80_F | TAATTGGCAAGGTGCAAG |
| KRT80_R | GGCCCTGATATTCCTCATAG |
| GALNT1_F | TCAAACATGACAGGAGAACAG |
| GALNT1_R | ACCCACCATAGGTCATATCAG |
| CALHM3_F | GGTGATGGTCGAGGAGTG |
| CALHM3_R | AGCACATGTACCTGATGATGC |
| MRPL33_F | CTCCGCGGTCTTCTTTGC |
| MRPL33_R | AGTCGGTTTCTCTTGGTGTTG |
| PGM2_F | CCTTTGTGCCCTTCACAG |
| PMG2_R | CTGCTTTGGATTGTGAGATG |
| THRIL_F | ATCCCCACAGCTGTCCACCT |
| THRIL_R | ACGATACACAGGTGCTTTTT |
| NEK10_F | GCAAACAACAGCTTCCAG |
| NEK10_R | TCAAGTTCAACAGCTTCTGTG |
| GTF3C6_F | GAAGAGGAGGAGCAGTTGG |
| GTF3C6_R | GAATGGGCCTCTCAGTGTC |
| GALNT3_F | ACCTCCTGAATGTATTGAAC |
| GALNT3_R | TATTGCAGGTGAAGAATAGAG |
| SH3TC2_F | GAACATTAATCCAGACCTGACAC |
| SH3TC2_R | CCTCCTGGTCCTCATTCTC |
| THRAP3_F | GGTAGGCCCAGAAGTGTATG |
| THRAP3_R | AGCACCACTGGCTTCTTC |
| RAB31_F | GGTCAGGAACGGTTTCATTC |
| RAB31_R | GCTCCTTGACCCATTTCTTC |
| AP1AR_F | AAAGCTCTTGGAGCAAGAAAG |
| AP1AR_R | CATTGTTGGAAGGATGATATTG |
| DDX21_F | AGCAAACCGAGGAGAAAGAG |
| DDX21_R | AGGAGAATTCATGTCAACTTCAG |
| LIF_F | TCTTGGCGGCAGGAGTTG |
| LIF_R | GTGACATGGGTGGCGTATG |
| CERK_F | GAAGCTGACGTCCAGACC |
| CERK_R | ATGGAGGCTAAGGTGAACAG |
| PAQR5_F | TGCCCTTCTGGTTCTTTG |
| PAQR5_R | GCTGTCATTCTTGATGTCTGTC |
| CHMP5_F | TGGCACGGTGGACAGTAG |
| CHMP5_R | GGACCCTCTCTCATCTTCTTG |
| COPS8_F | CCATAATGACATGAATAATGCAA |
| COPS8_R | TGAGCGTTGATGGTTGTATAG |
| CCDC65_F | ACAATGTCATCAAGTCTTTAGC |
| CCDC65_R | CTCCATGTTGTAACTTTCCTC |
| HYAL1_F | ATACAAGAACCAAGGAATCA |
| HYAL1_R | ACTGGTCACGTTCAGGAT |
| LY6D_F | CAAGCATTCTGTGGTCTG |
| LY6D_R | AGAGGCTCCACTGTGTTC |
| GRHPR_F | GCGGCAGACTGTGAGGTG |
| GRHPR_R | GGTCGGAGAGGAGGCAGAG |
| ANKRD13A_F | CAGGGCCAGAATGTGGAG |
| ANKRD13A_R | CAAATGTCCCAAGGAAACAG |
| SLC9A2_F | CCAAGATTGGCTTCCATC |
| SLC9A2_R | TATAAGAAGGCAGCTCTCAGG |
| SLK_F | ACAATCTTTGGATCCTCATTG |
| SLK_R | TTCAAGCATCACAGCATCTAC |
| EDEM1_F | GCCACGATAAGGGTCCTG |
| EDEM1_R | GCTGCTTGGAGTCAGTTATTATTC |
| SEC24D_F | GGAGCGGGAACAGACTTC |
| SEC24D_R | GCTGAGAATACGGAGGTGTAG |
| CISD1_F | AGCGTACGAGTTGAATGGAT |
| CISD1_R | TGTCTTTCTGGATGTGAAGGT |
| RNF38_F | ATGATCAGCTCCATCAAG |
| RNF38_R | TACTACAAGCAGGGATGTG |
| PROCR_F | CTCAGATGGCCTCCAAAG |
| PROCR_R | ATCGTGGTGTTGGTGTCTG |
| SLC26A9_F | TTGCTCACCCACTGCTTG |
| SLC26A9_R | CGAAGAGGGTAAGGGAGTATG |
| SPDEF_F | CAGGCAGCTAACAGACACAG |
| SPDEF_R | GGGATACGCTGCTCAGAC |
| LRG1_F | CCCAGTCCAGGCAGGTATAAG |
| LRG1_R | CCCCTGGGCTTTTTGGTC |
| SUMO2_F | AAGCCCAAGGAAGGAGTC |
| SUMO2_R | CGTTCACAATAGGCTTTCATTAG |
| CCRL2_F | CGTTTCTTAAAAGGCAGTCTGA |
| CCRL2_R | GCTCTCCAGTTCACCTTCTATG |
| LMCD1_AS1_F | AAACAGGAGAGCCGAGGGGACCT |
| LMCD1_AS1_R | AGGACCTCAGAGCTGGAGTGAGG |
| C6orf165_F | CAGAATCTTGTTAAGCTTT |
| C6orf165_R | CTCGATTCGTATAATTCA |
| CENPB_F | CGATCCTGAAGAACAAGCG |
| CENPB_R | CGATCCTGAAGAACAAGCG |
| CXCL8_F | GACAAGAGCCAGGAAGAAAC |
| CXCL8_R | AAACTGCACCTTCACACAG |
| TNNC1_F | CTACAAGGCTGCGGTAGAG |
| TNNC1_R | AGCTCCTTGGTGCTGATG |
| IL6R_F | CTTGCTGGTGGATGTTCC |
| IL6R_R | TTCCTCACCAAGAGCACAG |
| IER3_F | CTCGCGGGCACCGAAAGC |
| IER3_R | GCTGGCGCCGGACCACTC |
| C10orf91_F | TGGGAGGGCAGAGGACAGACCA |
| C10orf91_R | GTCTGGGGTTGGCCTTGAT |
| YIPF6_F | TTCGCAATACCATCATGC |
| YIPF6_R | CCCACAAATCCCAATCTC |
| CEACAM1_F | CTTCTGCTCACAGCCTCAC |
| CEACAM1_R | CAGATTGTGGACAAGGAGAAG |
| SPNS3_F | TCATCATTGCAGGAGTGC |
| SPNS3_R | CAAACACAGGTGCAGACAG |
| SMIM13_F | GACTCTGCTTGTGTTCGTG |
| SMIM13_R | AAATACCAACCGCACACC |
| SSTR5_AS1 | CTGCTGCCCTCGTCTACCAGTG |
| SSTR5_AS1 | CATGTTGCCCAGGCTGGTCTT |
| BUD13_F | ATTTGCCTGTGGTGGCAGAG |
| BUD13_R | TTGAGGGTAGGTCTTCGTTGTG |
| SLC5A5_F | ATTGTAGCCACGATGCTG |
| SLC5A5_R | GTCACTTGGTTCAGGATGAG |
| TCEAL3_F | CAGGTCTGCGCGTCTGTT |
| TCEAL3_R | TTTCCCCTCCTTTTCCTGTC |
| GBP3_F | GGGAAGAATAAGGGCTTCTC |
| GBP3_R | AAGCAGGACTAAGGTGTGTTC |
| TMEM30B_F | ATGACCTGGAGCGCCACGGCCC |
| TMEM30B_R | TGCCAGGCGGGGAGGC |
| LINC00920_F | GCTAAACATGGATCCCCTCTGAAA |
| LINC00920_R | AACGGGCTGCAAGGACTGAA |
| mt_ND1_F | CACCCAAGAACAGGGTTTGT |
| mt_ND1_R | TGGCCATGGGTATGTTGTTAA |
| 18S_rRNA_F | TAGAGGGACAAGTGGCGTTC |
| 18S_rRNA_R | CGCTGAGCCAGTCAGTGT |

**Supplementary**
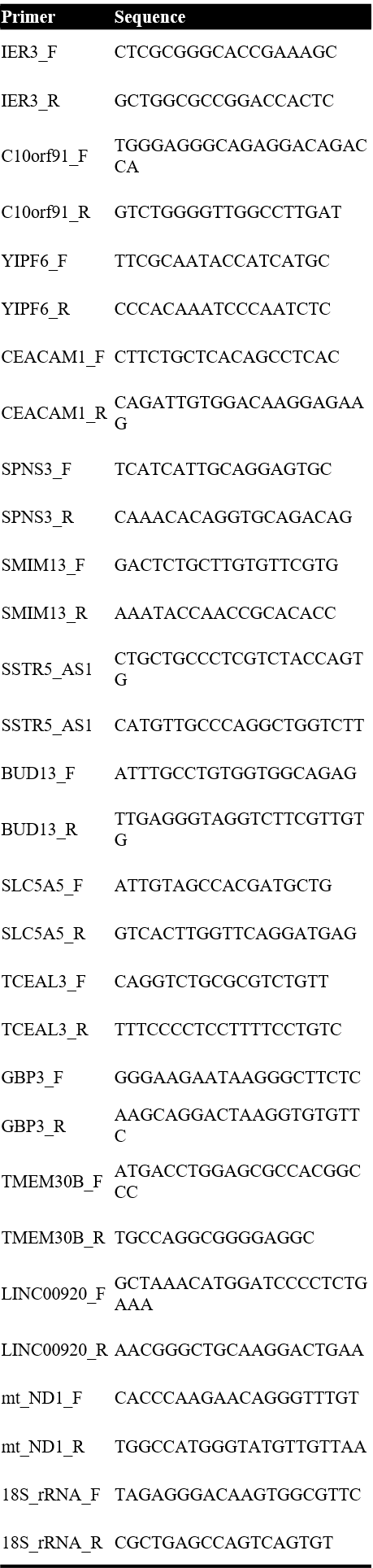
**Table S3.** Demographic characteristics for the SNP association study of Table 2.

| **Characteristics** | **Controls** | **All gastric cancer** | **Diffuse-type cases** | **Intestinal-type cases** |
| --- | --- | --- | --- | --- |
| Participants, n | 1,206 | 941 | 393 | 548 |
| Male | 678 (56.2%) | 646 (68.7%) | 420 (76.6%) | 226 (57.5%) |
| Female | 528 (43.8%) | 295 (31.3%) | 128 (23.4%) | 167 (42.5%) |
| Age, year | 55.3 ± 12.1 | 60.7 ± 12.2 | 63.9 ± 10.0 | 56.2 ± 13.5 |
